# Allosteric inhibition of RAN decreases miR-126 biogenesis in endothelial cells and controls acute myeloid leukemia growth

**DOI:** 10.1038/s42003-026-10026-0

**Published:** 2026-04-14

**Authors:** Melissa Valerio, Wenyuan Wei, Hyunjun Kang, Ning Ma, Supriyo Bhattacharya, Genevieve Baker, Weidong Hu, Lianjun Zhang, Dinh Hoa Hoang, Jia Feng, Hongyu Zhang, Bin Zhang, J. Jefferson P. Perry, Nagarajan Vaidehi, Le Xuan Truong Nguyen, Guido Marcucci

**Affiliations:** 1 Department of Hematologic Malignancies Translational Science, Beckman Research Institute and City of Hope National Medical Center, Duarte, CA USA; 2 Irell and Manella Graduate School of Biosciences, City of Hope, Duarte, CA USA; 3 Department of Computational and Quantitative Medicine, Beckman Research Institute of the City of Hope, Duarte, CA USA; 4 Department of Molecular Diagnostics & Experimental Therapeutics, Beckman Research Institute of City of Hope, Duarte, CA USA; 5 Beckman Research Institute, City of Hope National Medical Center, Duarte, CA USA; 6 Department of Hematology, Peking University Shenzhen Hospital, Shenzhen, China; 7 Early Detection and Prevention Division, Translational Genomics Research Institute, Phoenix, AZ USA; 8 School of Biomedical Engineering, International University, Vietnam National University, Ho Chi Minh City, Vietnam

**Keywords:** Acute myeloid leukaemia, Target validation, RNA

## Abstract

The small GTPase RAN plays a role in the biogenesis of mature miR-126, which is supplied by the bone marrow arterioles to leukemic stem cells (LSCs). MiR-126 supports the homeostasis of LSCs that initiate and maintain acute myeloid leukemia (AML). While therapeutic targeting of RAN has been difficult due to its structural features, through molecular dynamics simulations and docking studies, we have identified MAR-3.6.2 as a novel allosteric inhibitor that binds in a cryptic pocket in the C-terminal domain of RAN. We showed that MAR-3.6.2 disrupted RAN interaction with its guanine nucleotide exchange factor RCC1 and prevented the nuclear switch of RAN-GDP to RAN-GTP. This in turn led to RAN nuclear retention and reduced the RAN/XPO5-mediated export of pre-miR-126, thereby limiting mature miR-126 biogenesis in endothelial cells and their exogenous supply of mature miR-126 to LSCs. In a Mll^PTD/WT^/Flt3^ITD/ITD^ AML murine model, MAR-3.6.2 reduced leukemia burden, prolonged survival, and decreased LSC frequency in secondary transplants. These findings highlight MAR-3.6.2 and future, potential derivatives as a promising small molecule-based approach to eradicate AML LSCs via inhibition of RAN/XPO5 trafficking and block of miR-126 biogenesis.

## Abstract (web summary)

Allosteric inhibition of RAN disrupts miR-126 biogenesis and endothelial support of leukemic stem cells, reducing AML burden and prolonging survival, establishing a strategy to target LSC driven disease through RAN-dependent RNA trafficking.

## Introduction

RAN (RAS-related nuclear protein), a small GTPase of the RAS superfamily, is a dynamic protein equilibrating between two conformational states: active (RAN-GTP bound) and inactive (RAN-GDP bound)^1,2^. It plays a critical role in cancer by promoting proliferative signaling, apoptosis resistance, and metastasis, thereby establishing itself as a potential therapeutic target^1,2^. While small GTPases have traditionally been regarded as “undruggable,” recently a covalent allosteric inhibitor for the KRAS G12C mutant has paved the way for pharmacological inhibition of other small GTPases^3^. Small molecule inhibitors can either compete with GDP or GTP at the nucleotide-binding site or bind to distant allosteric sites to modulate RAN function. Given the high intracellular concentrations and strong binding affinities of guanosine nucleotides, allosteric inhibition—rather than direct competition with GDP or GTP—appears to be a more viable strategy for targeting RAN.

We have previously reported on a key role for RAN in the biogenesis of miR-126, a microRNA (miRNA) critical for regulating leukemic stem cell (LSC) homeostasis and activity^4,5^. In acute myeloid leukemia (AML), miR-126 is produced by arteriolar endothelial cells (ECs) and supplied by them to LSCs to maintain homeostasis of these cells and in turn to support disease growth^6,7^. Thus, targeting RAN-GTP may represent a promising approach for eliminating LSCs and curing AML.

MiRNAs are small non-coding RNAs that suppress protein expression by targeting messenger RNAs (mRNAs). They are transcribed as primary miRNAs (pri-miRNAs), which are processed by DROSHA into precursor miRNAs (pre-miRNAs) and then exported from the nucleus to the cytoplasm to be further processed by DICER into mature miRNAs. We previously reported that RAN-GTP forms a complex with XPO5 (also known as Exportin 5) in the nucleus, binding to pre-miR-126 and exporting it to the cytoplasm^4,5^. Upon GTP hydrolysis, pre-miR-126 is released from the RAN/XPO5 complex in the cytoplasm and processed into mature miR-126 (Fig. S1A, left). We previously reported that in the BM niche, in addition to their own endogenous production of miR-126, LSCs received an exogenous supply of this miRNA from arteriolar ECs^6,7^. Thus, we sought to investigate RAN as a therapeutic target in AML. Notably, analysis of the TARGET database revealed that AML patients with high RAN expression have significantly lower survival rates compared to those with low RAN expression (Fig. S1B, *p* < 0.0001). By identifying and characterizing allosteric inhibitors of RAN, we aim to inhibit its activity and disrupt the miR-126-mediated support of LSCs (Fig. S1A, right).

## Results and discussion

**Fig. 1:**
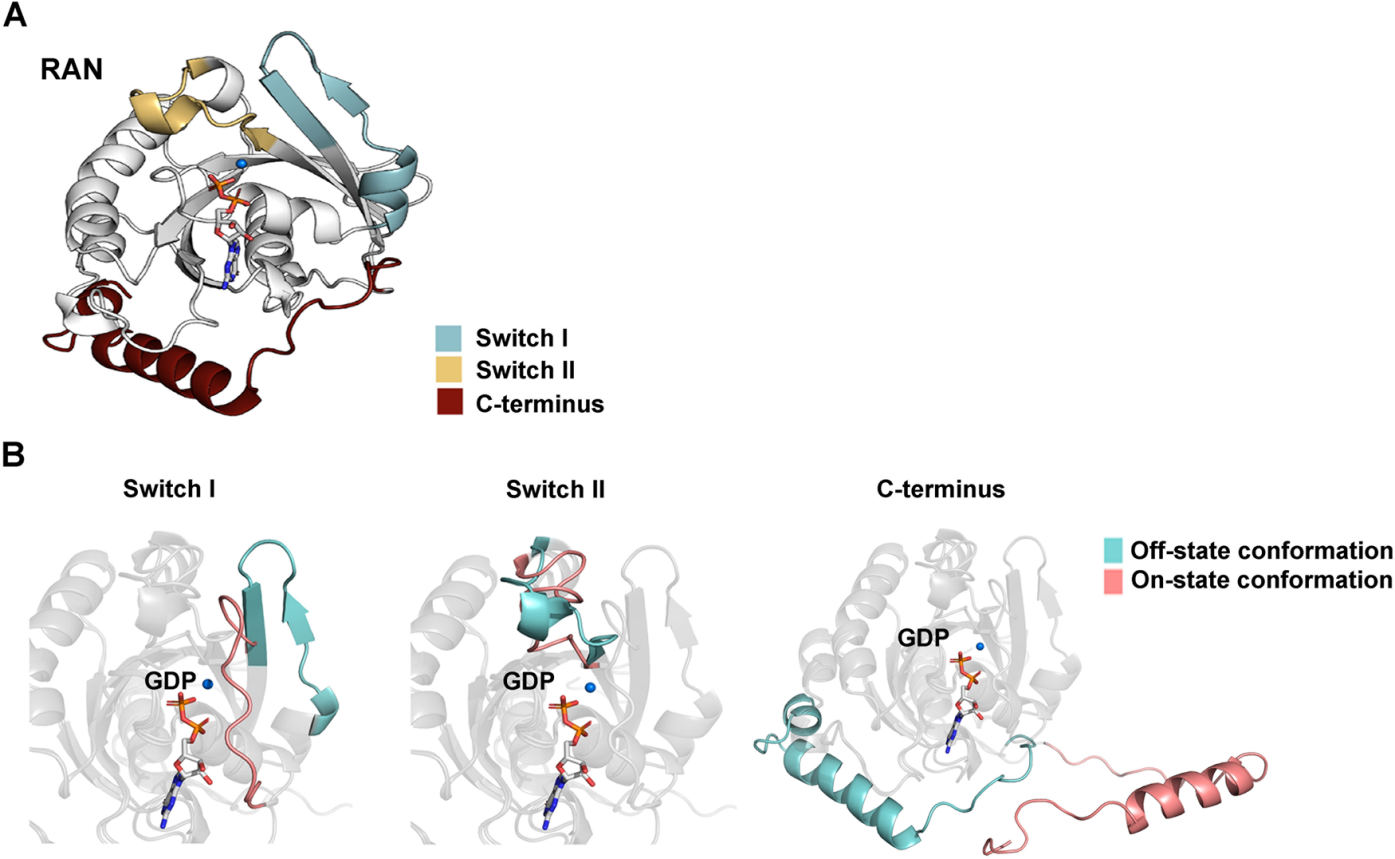
Molecular dynamics simulation analysis of RAN structure. **A** Structure of RAN protein highlighting switch I, switch II, and the C-terminal tail. **B** Schematic representation of conformational changes in switch I (left), switch II (middle), and C-terminal (right) regions of RAN in the active (GTP-bound, on-state) versus inactive (GDP-bound, off-state) conformations.

Unlike other members of the RAS superfamily whose C-terminal region is crucial for membrane association and signaling function, RAN lacks a C-terminal membrane-anchoring motif and instead possesses a unique disordered C-terminal region lacking a defined structure and existing in a flexible, dynamic, unstructured state (residues 180–216). This region features an acidic tip (210DEDDDL216) that plays a key role in autoregulating RAN’s conformational states and activity^8^. Three structural regions—switch I (residues 30–47), switch II (residues 65–80), and the C-terminal domain undergo conformational changes upon GTP binding (Fig. 1A). In the GDP-bound (inactive/off) state, the C-terminal region interacts with the core of RAN, stabilizing this state^1^. Upon transitioning to the GTP-bound (active/on) state, switch I region undergoes a translational movement, losing its secondary structure (Fig. 1B, left), while switch II rotates, repositioning the catalytic glutamine Q69 to face the nucleotide (Fig. 1B, middle). Additionally, the C terminus detaches from the RAN core and interacts with the RAN binding protein (RANBP) (Fig. 1B, right), enabling RAN to participate in nuclear transport and other regulatory functions.

**Fig. 2:**
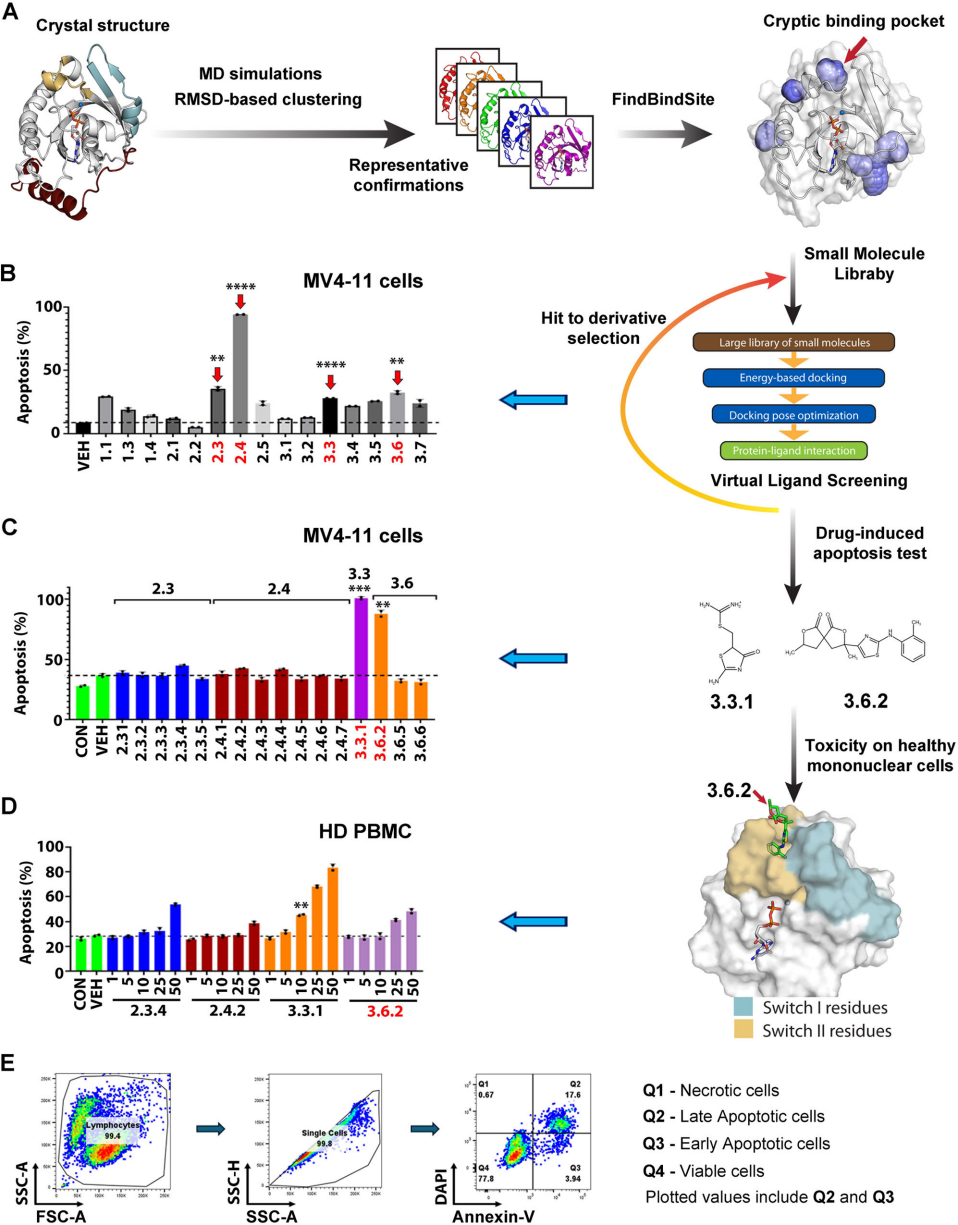
Identifying small molecules targeting RAN using MD simulations, VLS, and in vitro screening. **A** Top: Workflow of molecular dynamics (MD) simulation with RMSD-based clustering (left and middle), followed by FindBindSite analysis identifying a cryptic binding pocket on RAN (right). Right column: Virtual ligand screening (VLS) of small molecule library (second panel), molecular structure of the top two candidates-MAR-3.3.1 and MAR-3.6.2 (third panel), and docking model of MAR-3.6.2 into the RAN structure (fourth panel). **B** Effects of small molecules from the first VLS screen on apoptosis of MV4-11 cells. MV4-11 cells were treated with each indicated molecule (100 µM), and apoptosis was measured and presented as Annexin-V+. **C** Effects of derivatives of hit molecules on apoptosis of MV4-11 cells. MV4-11 cells were treated with each indicated molecule (50 µM), and apoptosis was measured and presented Annexin-V+. **D** Effects of hit molecules on PBMCs isolated from healthy donor (HD) peripheral blood. HD PBMCs were treated in a dose-dependent manner with four analogs, including MAR-2.3.4, MAR-2.4.2, MAR-3.3.1, and MAR-3.6.2, for 24 h. **E**. Gating strategy for apoptosis analysis. Apoptosis was measured and presented as Annexin-V+. Data are mean ± SE, based on triplicate determinations, and presented in a bar graph.

To identify small molecule inhibitors targeting RAN, we employed Molecular Dynamics (MD) simulations to uncover cryptic binding sites that are allosteric to the GDP/GTP binding site and could serve as potential druggable pockets^9^. Given the dynamic and disordered nature of the C-terminal region of RAN, MD simulations were performed based on the three-dimensional structure of the GDP-bound (inactive/off) state for a total of 4 μs (Fig. 2A, top left). To confirm the convergence of the individual velocities, we calculated root-mean squared deviation (RMSD) of protein Cα atoms (Fig. S2A). Clustering of the conformational ensemble from MD simulations, using RMSD clustering revealed five distinct conformational clusters (Fig. 2A, top middle). A computational method, FindBindSite^10^, to identify putative small molecule binding sites, was applied to the five representative conformations extracted from these clusters. A cryptic pocket was identified between the switch I and switch II regions, representing a potential site for small molecule binding to inhibit RAN activity (Fig. 2A, top right and S2B).

To screen for potential RAN inhibitors, a Virtual Ligand Screening (VLS) workflow was developed using the Glide and Prime modules of the Maestro suite (Schrödinger, LLC, New York, NY, 2022). Fifteen small molecules from the ChemBridge core and express libraries were docked and refined through iterative filtering to identify optimal candidates (Fig. 2A, right side, second panel). Details on docking methods and refinement are provided in the Methods section. The top scoring 15 molecules were tested for cytotoxic activity in AML cells, with four compounds (2.3, 2.4, 3.3, and 3.6) inducing over 20% apoptosis in MV4-11 cells at 100 µM (Fig. 2B, E). Since 100 µM is a relatively high testing concentration, chemically similar analogs effective at lower concentrations were identified using chemical similarity searches in ChemBridge, MCule, PubChem, and Enamine libraries, yielding 1802 analogs (see “Methods”). Among 16 experimentally tested analogs, two compounds (MAR-3.3.1 and MAR-3.6.2) exhibited stronger activity, inducing over 60% apoptosis in MV4-11 cells at 50 µM (Fig. 2A, right side, third panel and 2C). Of these, MAR-3.6.2 demonstrated lower toxicity to healthy donor PBMCs (Fig. 2A, right side, fourth panel and 2D), identifying it as a potential candidate or, more likely a scaffold for further optimization studies.

**Fig. 3:**
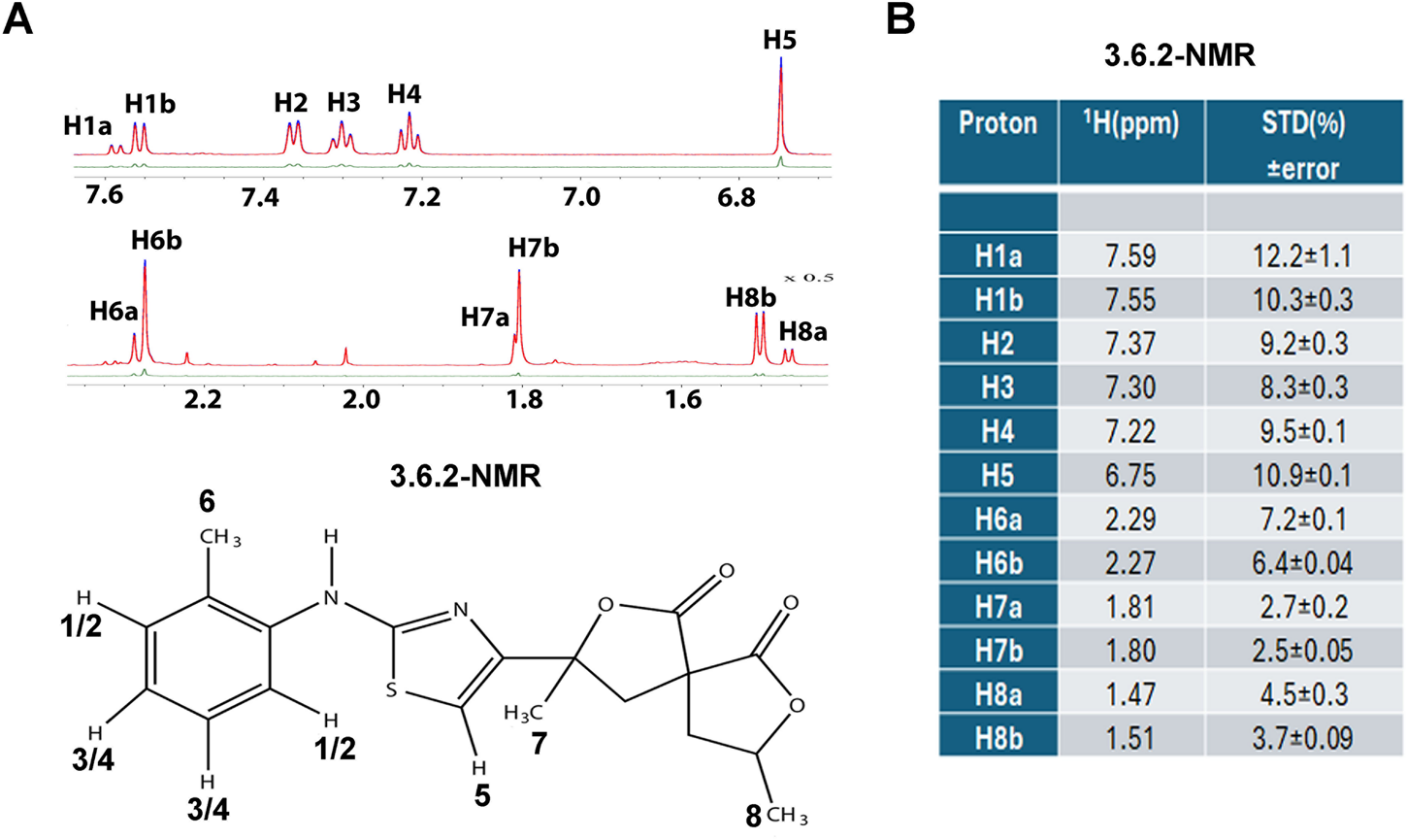
Saturation transfer difference (STD) analysis of RAN and MAR-3.6.2 interaction. **A** NMR analysis of MAR-3.6.2 binding to RAN, showing the 1D reference spectrum (blue), saturated spectrum (red), and difference spectrum (green), with five aromatic protons and three methyl groups **B** Proton assignment on the MAR-3.6.2 molecule for NMR analysis with a table showing STD values and corresponding chemical shift values.

Since NMR can detect weak binders to proteins, we used this approach to assess the potential direct binding of MAR-3.6.2 to RAN. Significant saturation transfer difference (STD) values observed in aromatic protons and a methyl group on the benzyl ring in the presence of GDP (see “Methods”) suggested a possible direct interaction between MAR-3.6.2 and RAN (Fig. 3A). The variation in STD values among different protons supported a specific drug-protein binding interaction, with protons from the minor diastereomer exhibiting higher STD values (Fig. 3B). Since the binding of MAR-3.6.2 was found in the presence of GDP, we postulate that this compound was an allosteric binder.

**Fig. 4:**
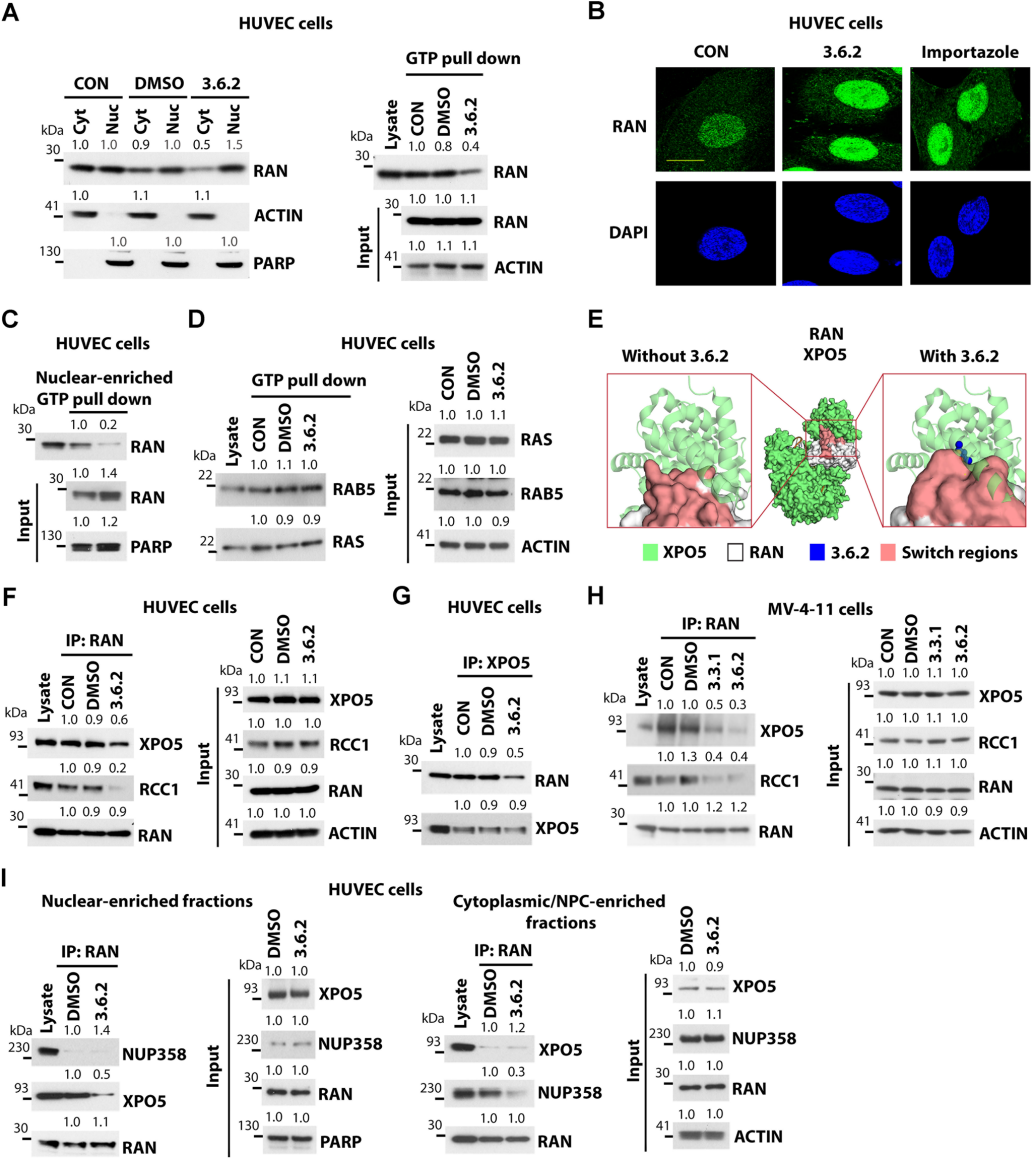
Effect of MAR-3.6.2 on RAN activities. **A** Effect of MAR-3.6.2 on RAN localization and GTP binding in HUVEC cells. Left, cells were treated with DMSO control or MAR-3.6.2 (25 µM) for 24 h, fractionated into cytoplasmic and nuclear fractions, and immunoblotted with anti-RAN antibody. Right, lysates from treated cells were subjected to GTP pull-down using GTP-conjugated agarose beads, followed by immunoblotting with anti-RAN antibody. Input loading controls are shown. **B** Effect of MAR-3.6.2 and Importazole on RAN localization in HUVEC cells. HUVEC cells were treated with DMSO control, MAR-3.6.2 (25 µM) for 24 h, or Importazole (10 µM) for 1 h and stained with an anti-RAN antibody. Scale bar, 10 μM. **C** Effect of MAR-3.6.2 on RAN-GTP levels in the nucleus of HUVEC ECs. Cells were treated with DMSO control or MAR-3.6.2 (25 µM) for 24 h, fractionated into nuclear fraction and lysate were subjected to GTP pull-down using GTP-conjugated agarose beads, followed by immunoblotting with anti-RAN antibody. Input loading controls are shown. **D** Effect of MAR-3.6.2 on other GTPases in HUVEC ECs. Cells were treated with DMSO control or MAR-3.6.2 (25 µM) for 24 h, lysates from treated cells were subjected to GTP pull-down using GTP-conjugated agarose beads, followed by immunoblotting with anti-RAB5 and anti-RAS antibodies. Input loading controls are shown. **E** Structure of the RAN-XPO5 crystal (PDB ID: 3A6P, left and middle) and alignment of MAR-3.6.2 with the crystal structure (right). The switch regions of RAN exhibit good complementarity with XPO5 (left), whereas significant steric clashes are observed in the RAN-MAR-3.6.2 complex (right), suggesting disrupted interaction. **F** Effects of MAR-3.6.2 on RAN binding with RCC1 and XPO5. HUVEC cells were treated with DMSO control or MAR-3.6.2 (25 µM) for 24 h. Lysates were immunoprecipitated with anti-RAN antibody and immunoblotted using anti-RCC1 and anti-XPO5 antibodies. Input loading controls are shown. **G** Effects of MAR-3.6.2 on XPO5 binding with RAN. HUVEC cells were treated with DMSO control or MAR-3.6.2 (25 µM) for 24 h as described in Fig. 4F. Lysates were immunoprecipitated with anti-XPO5 antibody and immunoblotted using anti-RAN antibodies. **H** Effects of MAR-3.6.2 on RAN binding with RCC1 and XPO5. MV4-11 cells were treated with DMSO control, MAR-3.3.1 or MAR-3.6.2 (25 µM) for 24 h. Lysates were immunoprecipitated with an anti-RAN antibody and immunoblotted using anti-RCC1 and anti-XPO5 antibodies. Input loading controls are shown. **I** Effects of MAR-3.6.2 on RAN interaction with XPO5 and NUP358 in nuclear-enriched and cytoplasmic/nuclear pore complex (NPC)-enriched fractions of HUVEC ECs. Cells were treated with DMSO or MAR-3.6.2 (25 µM) for 24 h, subjected to subcellular fractionation, and RAN was immunoprecipitated using an anti-RAN antibody, followed by immunoblotting with anti-XPO5 and anti-NUP358 antibodies. Input loading controls are shown.

Once activated by the guanine nucleotide exchange factor RCC1 in the nucleus, RAN-GTP binds to transporter proteins (e.g., XPO5) and exits the nucleus^11,12^. To this end, we observed that treatment with MAR-3.6.2 increased nuclear retention of RAN, an effect also observed with importazole (an indirect RAN transport inhibitor here used as a positive control)^13^ (Fig. 4A, left and 4B). MAR-3.6.2 also reduced RAN GTP binding in HUVEC ECs (Fig. 4A, right and 4C). Of note, MAR-3.6.2 had no measurable impact on the GTP-binding activity of related small GTPases, including RAS and RAB5 (Fig. 4D). These findings suggest that MAR-3.6.2 disrupted RAN interaction with RCC1 and trapped RAN-GDP in the nucleus, leading us to hypothesize that reduced RAN-GTP availability in turn impaired its binding to XPO5 and decreased nucleus-to-cytoplasm export of pre-miR-126 and mature miR-126 biogenesis.

**Fig. 5:**
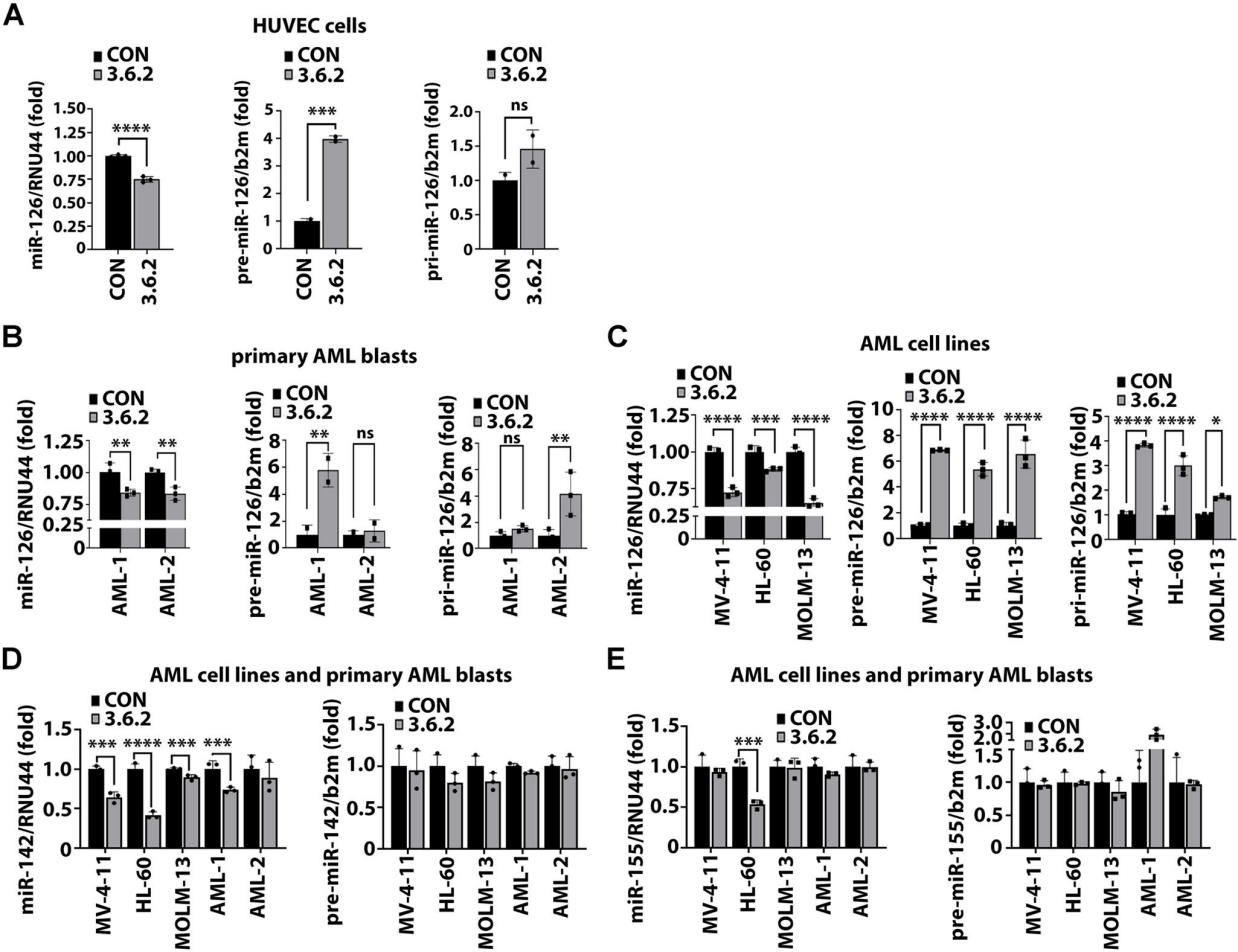
Effect of MAR-3.6.2 on miRNA biogenesis. **A** Effects of MAR-3.6.2 on miR-126 biogenesis. HUVEC cells were treated with DMSO control or MAR-3.6.2 (25 µM) for 24 h. Levels of miR-126 (left), pre-miR-126 (middle), and pri-miR-126 (right) were measured by qPCR. Data are mean ± SE, based on triplicate determinations, and presented in a bar graph. Effects of MAR-3.6.2 on miR-126 biogenesis. Primary AML blasts (**B**) and AML cell lines (**C**) were treated with DMSO control or MAR-3.6.2 (25 µM) for 24 h. Levels of miR-126 (left), pre-miR-126 (middle), and pri-miR-126 (right) were measured by qPCR. **D**,** E** Effects of MAR-3.6.2 on miR-142 and miR-155 biogenesis. AML cell lines and primary AML blasts were treated with DMSO control or MAR-3.6.2 (25 µM) for 24 h. **D** Levels of mature miR-142 (left) and pre-miR-142 (right) were measured by qPCR. **E** Levels of mature miR-155 (left) and pre-miR-155 (right) were measured by qPCR. Data are mean ± SE, based on triplicate determinations, and presented in a bar graph.

To this end, alignment of the RAN-XPO5 complex crystal structure (PDB ID: 3A6P) (Fig. 4E, left) with the structural model of MAR-3.6.2–bound RAN revealed significant steric clashes, suggesting the RAN-XPO5 interaction was disrupted (Fig. 4E, right). Immunoprecipitation assays confirmed that MAR-3.6.2 decreased the binding of RAN with RCC1 and of RAN with XPO5 in both HUVEC ECs and MV4-11 AML cells (Fig. 4F-4H). Cellular fractionation and immunoprecipitation assays further confirmed that MAR-3.6.2 reduced RAN–XPO5 binding in the nucleus and RAN–NUP358 binding in the cytoplasm, with NUP358 acting as a nuclear pore–associated RAN regulator (Fig. 4I). As we showed that RAN-XPO5 complex is implicated in miR-126 biogenesis, we then measured levels of pri- and pre-miR-126 and mature miR-126 as readout of functional impairment of RAN-XPO5 activity. We showed accumulation of pri- and pre-miR-126 and a decrease in mature miR-126 in MAR-3.6.2-treated HUVEC ECs, AML cell lines, and primary AML blasts, suggesting a decrease in the nucleus-to-cytoplasm export of pre-miR-126 for the final step of mature miR-126 biogenesis (Fig. 5A–C). Of note, we observed a modest decrease in mature miR-142 and miR-155, which are also involved in leukemogenesis^14,15^, without significant nuclear retention of the respective pre-miRs, in MAR-3.6.2–treated AML cell lines and primary AML blasts (Fig. 5D, E). Higher level of the nuclear retention pre-miR-126 levels and reduction of mature miR-126 were instead observed in MAR-3.6.2-treated leukemic cells (Fig. 5B, C), suggesting a more specific activity of this compound on miR-126 biogenesis.

**Fig. 6:**
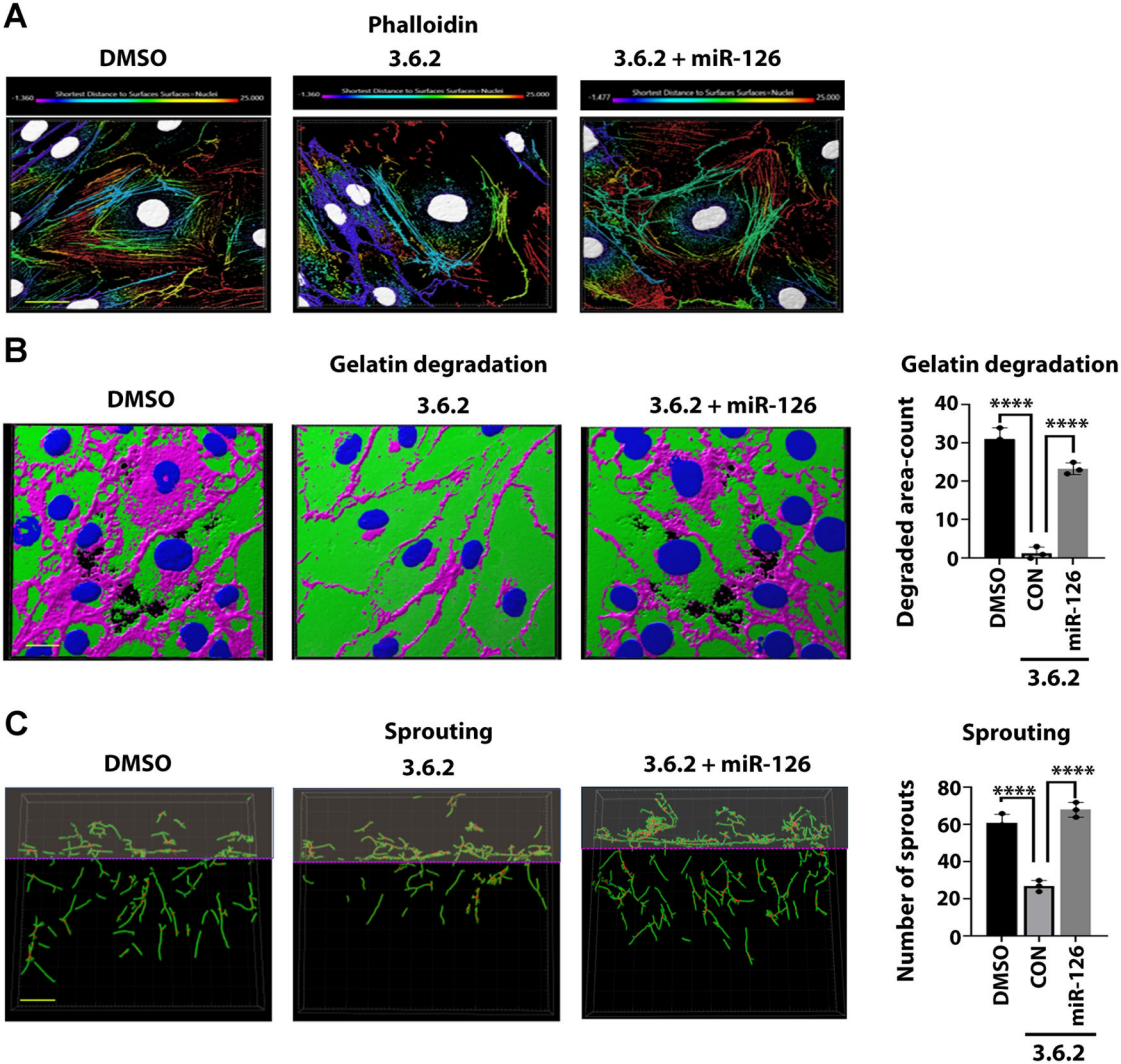
Effects of RAN inhibitor MAR-3.6.2 on endothelial cell activities. **A**–**C** Effects of MAR-3.6.2 on endothelial cell activities. HUVEC cells were treated with DMSO control, MAR-3.6.2 (25 µM) or MAR-3.6.2 (25 µM) and miR-126 mimic (2 µM) for 24 h. **A** Representative 3D images of phalloidin-stained cells. Scale bar, 20 μM. **B** Left, representative 3D images of gelatin degradation. Scale bar, 20 μM. Right, quantification of gelatin degradation. **C** Left, representative images of vascular sprout growth in a 3D matrix. 3D surfaces were rendered and analyzed using Imaris software. Scale bar, 200 μM. Right, quantification of vascular sprout growth in a 3D matrix.

MiR-126 is highly expressed in arteriolar ECs and we have previously reported a key functional interplay of these cells with LSCs to maintain leukemic homeostasis^4,6^. Thus, next we test MAR-3.6.2 on HUVEC ECs. We observed impaired EC activities as shown by demonstrated disruption of actin polymerization, as indicated by phalloidin immunostaining (Fig. 6A), reduction in gelatin degradation activity (Fig. 6B)^16^, and inhibiting 3D angiogenic sprouting (Fig. 6C) in MAR-3.6.2-treated HUVEC ECs compared to DMSO-treated controls. We postulated that these finding were due to reduction of miR-126 production, as we observed similar results when we treated HUVEC ECs with miRisten, an anti-miR-126 oligonucleotide (oligo) that decreases miR-126 levels and is currently in clinical trials (NCT07025564)^4,6,17^. MiRisten treatment impaired responses to chemoattractants (Fig. S3A), reduced wound healing activity compared to oligo scramble control (SCR) (Fig. S3B) and disrupted actin polymerization (Fig. S3C, D) and podosome formation (Fig. S3E), leading to decreased gelatin degradation activity (Fig. S3F) and inhibiting 3D angiogenic sprouting (Fig. S3G) similar to MAR-3.6.2. Conversely, miR-126 overexpression via miR-126 mimic treatment^6^ rescued the MAR-3.6.2–induced defects in actin polymerization, matrix degradation, and 3D angiogenic sprouting (Fig. 6A–C). Taken altogether, these data suggest that miR-126 plays a central and functionally essential role in mediating MAR-3.6.2–induced endothelial dysfunction and antileukemic activity.

**Fig. 7:**
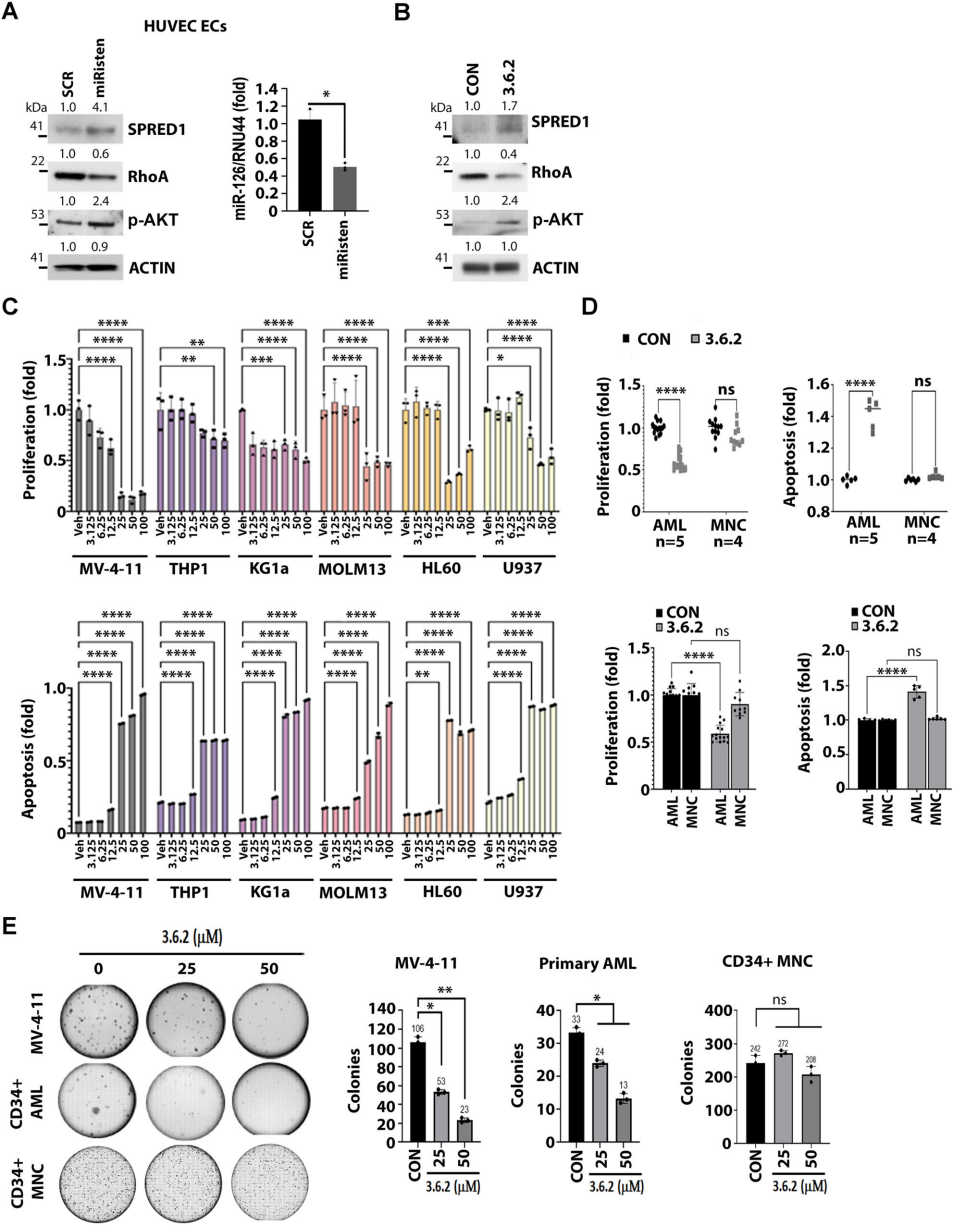
RAN inhibitor MAR-3.6.2 antileukemic activity in vitro. **A** HUVEC cells were treated with either SCR control or miRisten (2 µM) for 24 h. Left, protein levels of SPRED1, RhoA, and p-AKT were analyzed by immunoblotting. Right, levels of miR-126 were measured by qPCR. **B** HUVEC cells were treated with either DMSO control (CON) or MAR-3.6.2 (25 µM) for 24 h. Protein levels of SPRED1, RhoA, and p-AKT were analyzed by immunoblotting. **C**,** D** Effects of MAR-3.6.2 on AML cell line proliferation and apoptosis. AML cell lines (MV4-11, THP1, KG1a, Molm13, HL60, and U937) were treated with the indicated doses of MAR-3.6.2 for 24 h. **C** Cell proliferation was measured using a WST-1 assay and apoptosis was assessed by flow cytometry. **D** Effects of MAR-3.6.2 on proliferation and apoptosis of LSC-enriched AML blasts. CD34⁺CD38⁻ cells were isolated from primary MNCs (*n* = 4) or AML blasts (*n* = 5) and treated with DMSO control or MAR-3.6.2 (25 µM) for 24 h. Top left, cell proliferation levels. Top right, apoptosis levels indicated by Annexin-V+ cells. Bottom, comparison of effect of MAR-3.6.2 on primary LSC-enriched and HSC-enriched primary cells. **E** Effects of MAR-3.6.2 on colony formation of MV-4-11 cells, LSC-enriched AML blasts and HSC-enriched mononuclear cells. MV-4-11 cells, CD34⁺CD38^−^ AML blasts, and CD34^+^CD38^−^ mononuclear cells (2 × 10⁵ cells/mL, *n* = 3) were treated with DMSO control (CON) or indicated doses of MAR-3.6.2 for 24 h, then plated in methylcellulose. After 14 days, colonies were imaged under a light microscope (left) and counted (right). Data are mean ± SE, based on triplicate determinations, and presented in a bar graph.

The mechanisms through which decreased expression of miR-126 disrupted ECs function and angiogenesis^6^ were likely mediated by the increased levels of miR-126 targets SPRED1 and PIK3R2^7,18,19^. SPRED1 inhibits the RAS/MAPK pathway, reducing RhoA activity to restrain cytoskeletal reorganization, cell proliferation, and migration^20,21^. MiRisten treatment increased SPRED1 expression, lowering RhoA levels (Figs. 7A and S3C) and impairing actin cytoskeleton dynamics and migration in HUVEC ECs. PI3K, a heterodimeric lipid kinase composed of catalytic and regulatory subunits, promotes cell survival and migration by phosphorylating phosphoinositides (PIP) and initiating signaling cascades that regulate cytoskeletal dynamics^22^. PIK3R2, a gene in this pathway, encodes p85β, a regulatory subunit of class IA PI3K that modulates its activation and localization. Elevated PI3K activity—evidenced by increased downstream phosphorylated AKT (p-AKT) (Fig. 7A) and enhanced conversion of PIP substrates (Fig. S4A)—leads to PIP2 depletion and impairs the interaction between PIP2, Cortactin, and TKS5 (Fig. S4B), which is essential for podosome formation and matrix degradation in HUVEC ECs^23–25^. These signaling disruptions impair EC motility and structural integrity, underscoring miR-126’s role in cytoskeletal regulation. To this end, like miRisten, MAR-3.6.2 treatment decreased miR-126 levels (Fig. 5A), increased the expression SPRED1, reduced RhoA levels, and elevated p-AKT levels (Fig. 7B).

The observed inhibitory effects of MAR-3.6.2 on miR-126 expression and its relevance to LSC homeostasis prompted us to evaluate the therapeutic potential of this compound in AML^5,26^. miR-126 plays a critical role in supporting AML and LSC growth. We have previously reported that disruption of miR-126—either by directly inhibiting endogenous miR-126 in AML cells or by blocking its transfer from ECs—significantly impairs AML and LSC proliferation and induces apoptosis^4–6^. Notably, direct treatment with MAR-3.6.2 demonstrated dose-dependent inhibition of cell proliferation and induction of apoptosis across multiple AML cell lines (Fig. 7C). In primary CD34 + CD38 − AML blasts (enriched for LSCs), MAR-3.6.2 significantly inhibited proliferation and induced apoptosis within 24 h, whereas normal CD34⁺CD38⁻ mononuclear cells (enriched for HSCs) showed only a trend for modest reduction in proliferation and viability (Fig. 7D). Consistently, colony-forming assays demonstrated that MAR-3.6.2 treatment markedly suppressed colony formation in MV4-11 cells and CD34⁺ AML blasts, while exerting only a minimal, non-significant effect on normal CD34⁺ mononuclear cells (Fig. 7E). Although the inhibitory effects on normal CD34⁺ hematopoietic cells were modest and did not reach statistical significance, it is important to underscore that any degree of perturbation of normal hematopoiesis may have potential clinical implications and therefore it will require careful evaluation in future pharmacologic and toxicologic studies to translate RAN-inhibitors into the clinic. Overexpression of the GTP-binding RAN mutant Q69L rescued the MAR-3.6.2–induced inhibition of colony formation in both MV4-11 cells and CD34⁺ AML blasts (Fig. S4C).

To test whether MAR-3.6.2 disrupts EC-to-AML miR-126 transfer and the interplay of ECs with LSCs, HUVEC ECs were pretreated with MAR-3.6.2 and co-cultured with primary CD34⁺ AML cells. Compared with vehicle-treated EC controls, co-culture with MAR-3.6.2–pretreated ECs resulted in significantly reduced miR-126 levels in CD34⁺ AML cells, accompanied by decreased AML cell proliferation and colony formation. These results demonstrate that MAR-3.6.2–mediated suppression of miR-126 in ECs limits its transfer to AML cells and thereby inhibits AML cell growth (Fig. S4D).

**Fig. 8:**
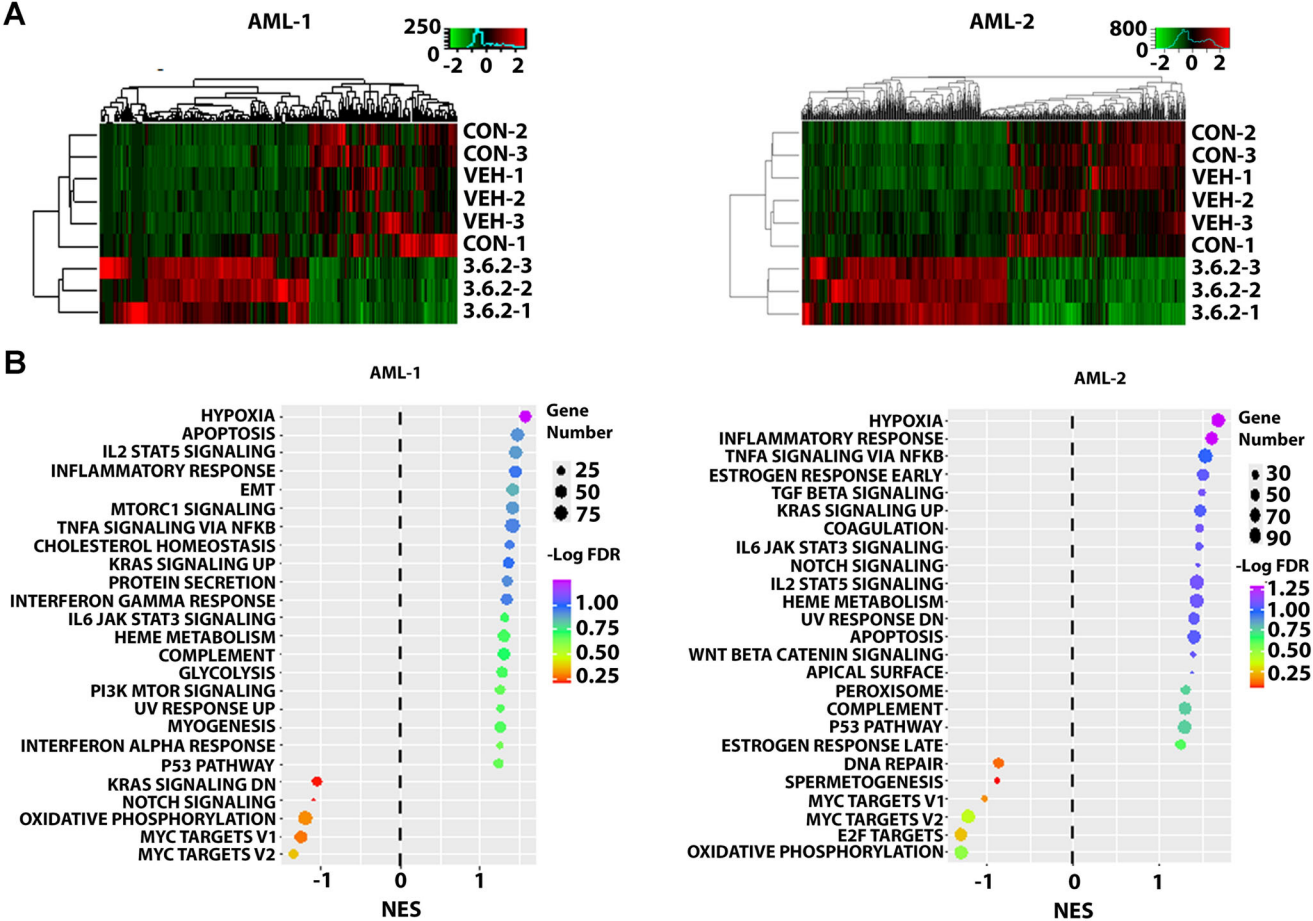
Effects of RAN inhibitor MAR-3.6.2 on gene expression in primary AML cells. **A**,** B** Primary AML cells were treated with DMSO control (VEH) or MAR-3.6.2 (25 µM) for 24 h. mRNA was extracted and subjected to RNA sequencing. **A** Unsupervised hierarchical clustering analysis revealed significant changes in gene expression, represented in the heatmap. **B** Gene set enrichment analysis results are shown in a dot plot with FDR-corrected statistical significance.

RNA-seq analysis of primary CD34 + AML blasts treated with MAR-3.6.2 (50 µM) revealed differential gene expression profiles (AML-1, 425 upregulated, 267 downregulated; AML-2, 1381 upregulated, 1210 downregulated; Fig. 8A). Upregulated genes were linked to apoptosis and inflammation, while downregulated genes were associated with oxidative phosphorylation and MYC targets reportedly support LSC homeostasis and activity (Fig. 8B).

**Fig. 9:**
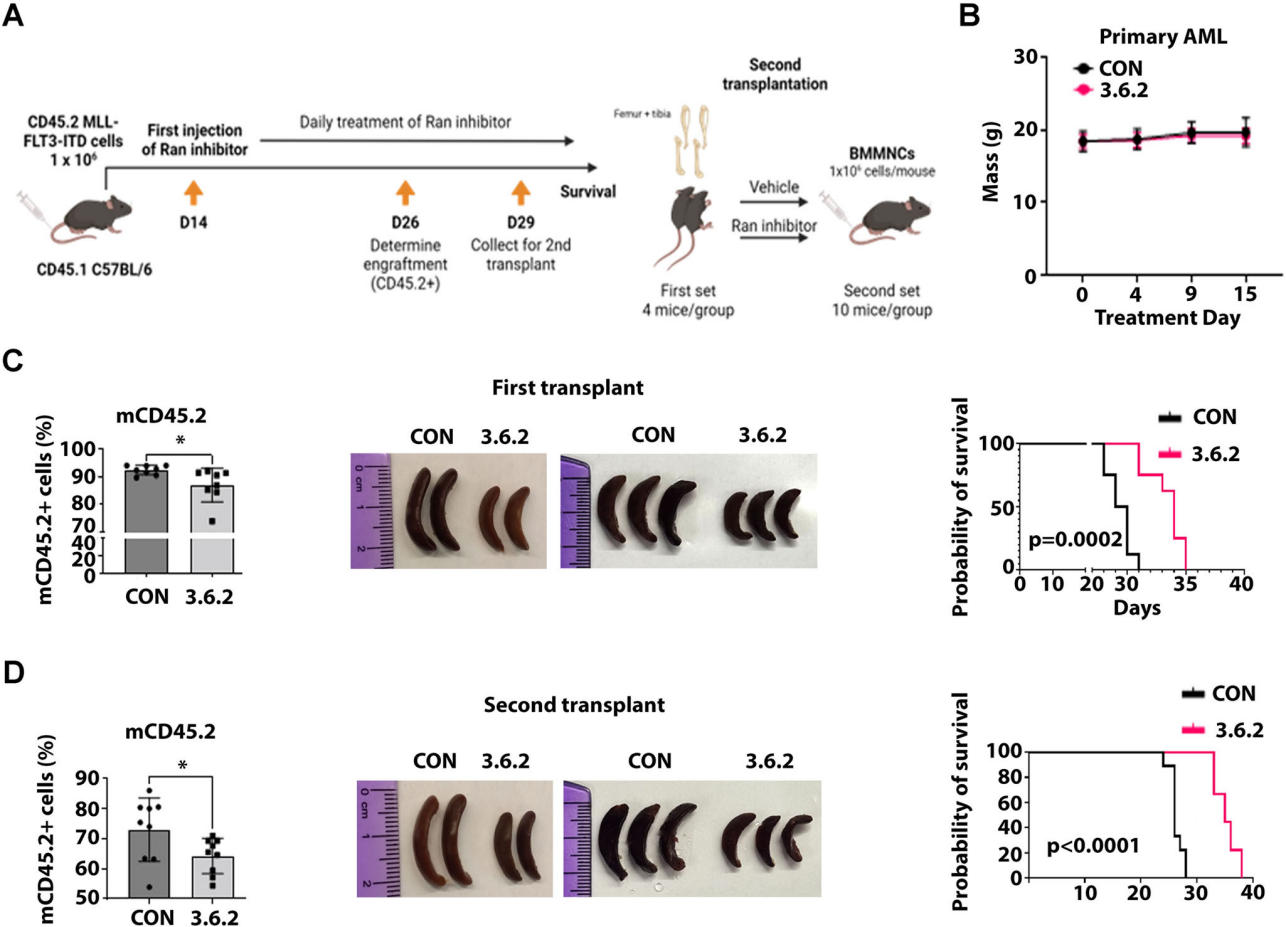
RAN inhibitor MAR-3.6.2 antileukemic activity in vivo. **A** Schematic of the experimental design. CD45.2 Mll^PTD/WT^/Flt3^ITD/ITD^ AML cells (1 × 10⁶/mouse) were transplanted into cohorts of wild-type B6 syngeneic mice. After 2 weeks, mice were treated with vehicle control or MAR-3.6.2 (25 mg/kg, IV, daily for 3 weeks). Eight mice were monitored for survival, and four mice were sacrificed three days after the last dose of treatment for AML engraftment analysis. Bone marrow cells were subsequently transplanted into secondary recipients (each group, *n* = 9). Created in BioRender. Valerio, M. (2026) https://BioRender.com/qcsn9nl**B** In vivo safety assessment of MAR-3.6.2. Body weight of treated mice was monitored throughout the study, with no significant changes observed over time. **C**,** D** Antileukemic effects of MAR-3.6.2 in vivo. **C** Primary transplantation model. Mll^PTD/WT^/Flt3^ITD/ITD^ CD45.2 AML cells (1 × 10⁶/mouse) were transplanted into cohorts of wild-type CD45.1 B6 mice. After 7 days, mice were treated with vehicle control or MAR-3.6.2 (25 mg/kg, IV, daily for 3 weeks) (*n* = 12 per group). Left, frequency of mCD45.2 AML cells in PB from control (CON)-treated (*n* = 8, mean 92.6%) vs. MAR-3.6.2-treated mice (*n* = 8, mean 86.2%) 21 days post-transplant, (3.6.2 vs. CON, *p* = 0.005). Middle, representative images of spleens from CON- vs. MAR-3.6.2-treated mice. Right, Kaplan-Meier survival curve of primary transplant recipients with Mll^PTD/WT^/Flt3^ITD/ITD^ AML treated with CON [*n* = 8, median survival (ms) 29 days] or MAR-3.6.2 (*n* = 8, ms 34 days). Statistical significance was determined using the Log-rank (Mantel-Cox) test. **D** Secondary transplantation model. BM cells from primary transplant recipients were collected and transplanted into secondary recipient mice. Left, frequency of mCD45.2 AML cells in PB of second transplant recipients treated with CON (*n* = 9, mean 73.0%) or MAR-3.6.2 (*n* = 9, mean 64.2%), (CON vs. 3.6.2, *p* = 0.04). Engraftment was analyzed at 2 weeks post-transplantation. Middle, representative images of spleens from second transplant recipients treated with control vs. MAR-3.6.2. Right, Kaplan–Meier survival curve of second transplant recipients treated with control (*n* = 9, ms 26 days) or MAR-3.6.2 (*n* = 9, ms 35 days), (3.6.2 vs. CON, *p* < 0.0001). Statistical significance was determined using the Log-rank (Mantel-Cox) test.

In vivo, MAR-3.6.2 exhibited significant antileukemic activity in the Mll^PTD/WT^/Flt3^ITD/ITD^ AML mouse model^27,28^, with no adverse effects on body weight (Fig. 9A, B). Mice treated with MAR-3.6.2 (25 mg/kg, IV, daily for 3 weeks) showed reduced leukemia burden, evidenced by a lower percentage of mCD45.2 cells in peripheral blood (CON: 92.6% vs. 3.6.2: 86.2%, *p* = 0.005), decreased spleen size, and extended median survival compared to vehicle-treated control (CON: 29 days vs. 3.6.2: 34 days, *p* = 0.0002) (Fig. 9C). Furthermore, MAR-3.6.2 reduced LSC burden, as indicated by a lower leukemia burden (mCD45.2+, CON: 73.0% vs. 3.6.2: 64.2%, *p* = 0.04), smaller spleen, and prolonged survival in secondary transplants of BM MNCs from MAR-3.6.2-treated donors compared to vehicle-treated donors (CON: 26 days vs. 3.6.2: 35 days, *p* < 0.0001) (Fig. 9D).

In summary, we identified MAR-3.6.2, a small-molecule allosteric inhibitor targeting the RAN GTPase, and established a systematic approach for identifying druggable allosteric binding sites, which can be extended to other challenging protein targets. Allosteric inhibition of RAN disrupted miR-126 expression and function in both endothelial and AML cells, leading to significant antileukemic effects. Although the effective in vitro concentrations of MAR-3.6.2 are currently in the micromolar range, this compound represents a first-generation chemical probe designed to validate the therapeutic relevance of allosteric RAN inhibition in the RAN–miR-126 axis. Ongoing structure-guided medicinal chemistry optimization aims to improve its potency, solubility, and overall drug-like properties, while pharmacokinetic and bioavailability studies are planned as part of its preclinical development. Even if MAR-3.6.2 cannot be further optimized, these compelling preliminary results support its use as a scaffold for the design of RAN inhibitors with more favorable pharmaceutical properties. In parallel, future translational strategies may include combination approaches with established AML therapies such as venetoclax or hypomethylating agents (HMAs) to enhance antileukemic efficacy at lower systemic exposures and improve clinical feasibility. While further optimization of MAR-3.6.2 is ongoing, these results provide compelling evidence that targeting RAN through allosteric inhibition represents a promising therapeutic strategy for AML.

## Methods

### An extensive description of the methods can be found in the Supplemental Methods

#### Human samples

AML bone marrow (BM) samples were collected from donors and patients at City of Hope National Medical Center (COHNMC) under one of four Institutional Review Board (IRB)-approved banking protocols (#06229, #03162, #07047, or #18067). Sample collection complied with Department of Health and Human Services (HHS) regulations and adhered to the Declaration of Helsinki. Written informed consent was obtained from donors (#06229) and patients (#03162, #07047, #18067) before sample collection. Patient characteristics of primary AML samples are detailed in Supplemental Table 1.

### Colony forming assay

The colony-forming potential of leukemic stem cells (LSCs) and hematopoietic stem cells (HSCs) was evaluated using a colony formation assay. Cells (5 × 10³) were treated with either small molecule MAR-3.6.2 (25 or 50 µM) or DMSO, then mixed with 1.5 mL of H4434 MethoCult (Stem Cell Technologies) and vortexed thoroughly. The cell mixture was plated in a 6-well plate and incubated at 37 °C. Colony formation was assessed between days 12 and 14 of culture.

### Mice

All transplants were conducted via retro-orbital intravenous (i.v.) injection. Mll^PTD/WT^/Flt3^ITD/ITD^ knock-in AML cells were injected into sublethally irradiated (4.5 Gy, XRAD 320-Precision X-Ray) C57BL/6 female mice (6–8 weeks old). In vivo treatments, including vehicle (VEH) or MAR- 3.6.2 (3.6.2), were administered daily via i.v. injection starting 7 days post-transplant for a total of 21 days. On day 26 post-transplant, mice were subjected to isoflurane anesthesia and peripheral blood was collected via retro-orbital blood collection for flow cytometric analysis of leukemic engraftment and complete blood count (CBC) assessment. Following treatment completion, four mice per group were humanely euthanized via CO_2_ inhalation, and bone marrow cells were extracted from the femur and tibia, pooled, and transplanted into secondary recipient mice.

All animals were housed in an AAALAC-accredited facility, and all experimental procedures were performed in accordance with federal and state regulations, as well as institutional protocols approved by the City of Hope Institutional Animal Care and Use Committee (IACUC; Protocol #22043). We have complied with all relevant ethical regulations for animal use.

### Isolation of mononuclear cells from patient samples

Each patient specimen was transferred into a 50 mL conical tube and diluted to 25 mL with pre-warmed 1× Dulbecco’s phosphate-buffered saline (DPBS) containing 2% fetal bovine serum (FBS). The sample was then gently layered over 20 mL of Ficoll-Paque Plus in a separate 50 mL conical tube and centrifuged at 300 × *g* for 32 min without braking. Following centrifugation, the peripheral blood mononuclear cell (PBMC) and plasma layer was carefully collected into a fresh 50 mL tube, diluted to 50 mL with warm 1× DPBS, and subjected to a second centrifugation at 2400 rpm for 8 min. After removing the supernatant, the cell pellet was resuspended in 10 mL of warm 1× DPBS, and cell count and viability were assessed. The processed samples were then frozen for subsequent analysis.

### Cell cultures and chemicals

U937, THP-1, Molm13, HL-60, KG-1a, and MV-4-11 cell lines were purchased from the American Type Culture Collection (ATCC) and cultured in IMDM (Iscove’s Modified Dulbecco’s Medium) or RPMI (Roswell Park Memorial Institute) medium supplemented with 10% fetal bovine serum (FBS) and 100 units/mL of penicillin/streptomycin. Cells were maintained at 37 °C in a humidified atmosphere with 5% CO₂. Human cell lines obtained from ATCC more than 6 months before manuscript submission, or those not cryopreserved at an early passage, were authenticated using ATCC’s human short tandem repeat (STR) DNA profiling service. Cell morphology was routinely monitored, and mycoplasma testing was performed regularly using a mycoplasma detection kit (Roche, Germany).

For small-molecule screening, compounds were purchased from VitasM (Champaign, IL), MCule (Palo Alto, CA), and ChemBridge (San Diego, CA) and dissolved in DMSO before use.

### Cell proliferation assay

The impact of small molecule inhibitors on AML cell proliferation was evaluated using the WST-1 assay (Cat #5015944001, Millipore Sigma). Cells were seeded at a density of 10,000 cells per well in a 96-well plate and treated with varying concentrations of inhibitors for 24 h at 37 °C. Following treatment, 10 µL of WST-1 reagent was added to 100 µL of culture medium, and the plate was incubated for an additional 2–3 h at 37 °C. The resulting formazan product, generated by WST-1 metabolism, was quantified using a multi-well spectrophotometer at 450 nm.

### RT-PCR and q-PCR analysis

Total RNA, including miRNA, was extracted using the AllPrep DNA/RNA/miRNA Universal Kit (Qiagen, Cat# 80224) according to the manufacturer’s instructions. Reverse transcription was performed using the TaqMan® MicroRNA Reverse Transcription Kit (Thermo Fisher Scientific, Cat# 4366596) with gene-specific primers for pre-miR-126 (042373250), pri-miR-126 (03303230), B2M (00187842), miR-126 (002228) and RNU44 (001094) from the TaqMan® MicroRNA Assay. Quantitative PCR (qPCR) was conducted using the TaqMan™ Universal Master Mix II, with UNG (Thermo Fisher Scientific, Cat# 4440038). Gene expression levels were normalized to internal reference genes using the Ct method, and relative expression was calculated as 2^−ΔCt^.

### Immunoblotting (IB) and Immunoprecipitation (IP)

Cells were washed with ice-cold PBS and lysed on ice for 30 minutes in RIPA buffer supplemented with 10 mM protease inhibitor cocktail (Thermo Fisher Scientific, Cat# 78429). The lysate was then centrifuged at 12,000 × *g* for 15 min at 4 °C, and the supernatant was transferred to a fresh Eppendorf tube.

For immunoprecipitation (IP), 500 µg to 1 mg of total protein lysate was incubated overnight at 4 °C with 2 µg of the indicated antibody per IP reaction. The next day, 50 µL of protein A/G agarose beads (Cell Signaling Technology, Cat# 9863; Cat# 37478) were added to the supernatant and incubated at 4 °C for 3 h. The beads were then washed five times with lysis buffer, resuspended in Laemmli Sample Buffer (Bio-Rad, Cat# 1610737EDU), and boiled at 95 °C for 10 min.

For immunoblotting, 2× Laemmli sample buffer (Bio-Rad, Cat# 1610737) was added to the lysate, and SDS samples were boiled at 95 °C for 10 min. Samples were loaded onto NuPAGE 4–12% Bis-Tris gels (Thermo Fisher Scientific, Cat# NP0322), separated by SDS-PAGE, and transferred onto a nitrocellulose membrane (Bio-Rad, Cat# 1620112) using Tris-Glycine transfer buffer. Following transfer, the membrane was washed with TBST (10 mM Tris-HCl, pH 8.0, 150 mM NaCl, 0.05% Tween 20) and blocked with 5% BSA. The membrane was then incubated with the primary antibody, followed by the appropriate secondary antibody. Protein detection was performed using SuperSignal™ West Femto Maximum Sensitivity Substrate (Thermo Fisher Scientific, Cat# 34096). Details of the antibodies used are provided in Supplementary Table S2.

### Assessment of apoptosis using flow cytometry

Apoptosis was assessed using Annexin-V and DAPI double staining followed by flow cytometry. Cells were harvested, washed twice with Annexin-V binding buffer (BD Bioscience, San Jose, CA), and resuspended in 100 μL of the same buffer containing Annexin-V APC (BD Bioscience, San Jose, CA). After incubation in the dark at room temperature for 15 min, cells were washed again and resuspended in 300 μL of binding buffer. DAPI (Sigma-Aldrich) was added immediately before analysis using an LSR II flow cytometer (BD Bioscience, San Jose, CA).

### Cell isolation and flow cytometry

Murine peripheral blood was collected via retro-orbital bleeding. RBCs were lysed with ACK Lysis Buffer (Gibco Cat# A1049201). For fluorescence-activated cell sorting (FACS) analysis, cells were incubated with fluorescently labeled antibodies in PBS with 0.5% BSA for 15 min at 4 °C. Flow cytometry was performed with a 5-laser BD LSRFortessa™ X-20 cell analyzer. Antibodies, such as mCD45.2 (Cat# 109814) were sourced from BioLegend. Data analysis was conducted using FlowJo software version 10.6.1.

### Statistics and reproducibility

To compare the means of 2 groups, data were analyzed using an unpaired, two-tailed Student’s t-test, unless otherwise stated. Results represent at least 2 independent experiments with triplicate determination, unless otherwise stated. Data are presented as mean ± standard error (SE), as indicated. *P* < 0.05 was considered statistically significant. Significance is denoted as follows: ns = not significant; *, *p* ≤ 0.05; **, *p* ≤ 0.01; ***, *p* ≤ 0.001; ****, *p* ≤ 0.0001. All statistical analyses were conducted using SigmaPlot 12.5 (Systat Software, Chicago, Illinois). All statistical tests were two-sided.

#### Ethical approval and consent to participate

AML samples used in this study were obtained from patients undergoing treatment at the City of Hope. Normal bone marrow and mobilized peripheral blood stem cells (PBSCs) were collected from healthy donors (HL). All sample acquisitions followed protocols that included obtaining written informed consent from participants, in compliance with the Department of Health and Human Services (HHS) guidelines and the Declaration of Helsinki. Written informed consent was obtained under approved banking protocols from donors (#06229) and patients (#03162, #07047, #18067) prior to sample collection. The study protocols were reviewed and approved by the City of Hope Institutional Review Board (IRB), in accordance with an assurance filed with and approved by HHS. Patient sample characteristics are detailed in Table S1. For animal studies, all mice were maintained in an Association for Assessment and Accreditation of Laboratory Animal Care (AAALAC) accredited facility, and all experimental procedures adhered to federal and state government guidelines as well as institutional protocols approved by the Institutional Animal Care and Use Committee at City of Hope (IACUC #22043).

### Reporting summary

Further information on research design is available in the Nature Portfolio Reporting Summary linked to this article.

## Supplementary information

Supplementary Information

Description of Additional Supplementary Files

Supplementary Data 1

Reporting Summary

## Data Availability

The RNA-seq data reported in this article has been deposited in NCBI’s Gene Expression Omnibus (GEO) and is accessible through GEO Series accession number GSE324546. All molecular dynamics simulation trajectories, topology files, and analysis scripts generated in this study are available at the MDRepo website, https://mdrepo.org, through the ID number MDR00021342. Source data, including uncropped western blots and numerical values, are provided in Supplementary Data 1. All other datasets generated during this study are available from the corresponding author on reasonable request.
