# Supplementary material for: Allosteric inhibition of RAN decreases miR-126 biogenesis in endothelial cells and controls acute myeloid leukemia growth
Source: Commun Biol. 2026 Apr 14;9:791. doi: 10.1038/s42003-026-10026-0 (PMC13254346; doi:10.1038/s42003-026-10026-0)
Supplement: Supplementary file 1 — Supplementary Information [file 42003_2026_10026_MOESM1_ESM.pdf]

SUPPLEMENTAL INFORMATION

1> Supplementary Materials and Methods (Page 2 to Page 8)

2> Supplementary Reference (Page 9)

3> Supplementary Tables: (Page 10 to Page 12)

Table S1. Characteristics of patient samples

Table S2. List of antibodies used for flow cytometry, immunoblotting, immunoprecipitation, and immunofluorescence analysis

Table S3. Molecular dynamics simulation setup detail

4> Supplementary Figure and Legends (Page 13 to Page 21)

Figure S1. Effects of RAN on regulation of miR-126 biogenesis

Figure S2. Cryptic binding pocket of RAN protein.

Figure S3. Effects of miRisten on endothelial cell activities

Figure S4. Anti-leukemic effect of RAN inhibitor MAR-3.6.2 in vitro

Materials and Methods

Starting structure and molecular dynamics simulation

There are two available inactive Ran structures (PDBID 3GJ0 and 2MMC). 2MMC is an NMR structure without GDP resolved, and 3GJ0 does not have the full C terminus resolved; both are not ideal for MD simulations. To circumvent those issues, we created our starting structure by grafting the GDP and Magnesium ion from 3GJ0 to 2MMC after aligning both structures in PYMOL (Cα RMSD: 0.24Å). We capped the termini of the structures using N-methyl group on the N-terminus and acetyl group on the C-terminus and subsequently energy minimized using conjugate gradient method with a convergence cutoff of 0.1kcal/mol/Å in Schrodinger Maestro suite (https://www.schrodinger.com/products/maestro). Input files for molecular dynamics simulations were generated using CHARMM-GUI[1]. Each Ran protein was solvated in explicit TIP3P water molecules in a cubic box (7.5nm x 7.5nm x 7.5nm) with 0.15M of potassium chloride for maintaining the physiological condition. We used software GROMACS[2] (Version 2021.3) with all-atom CHARMM36m force field[3] to perform molecular dynamics (MD) simulations. The specifics of the starting setup can be found in Table S3. MD simulations were performed at 310 K coupled to a temperature bath with a relaxation time of 0.1ps[4]. Pressure of the systems was calculated with molecular virial and was held constant by a weak coupling to a pressure bath with a relaxation time of 0.5ps. Equilibrium bond length and geometry of water molecules were constrained using the SHAKE algorithm[5]. The short-range electrostatic and van der Waals interactions were estimated every 2fs using a charged group pair list with cutoff of 8Å between centers of geometry of charged groups. Long-range van der Waals interactions were calculated using a cutoff of 14Å and long-range electrostatic interactions were treated with the particle mesh Ewald method[6]. Temperature was kept constant at 310K by applying the Nose-Hoover thermostat[7]. Desired pressure for all systems were achieved by using Parrinello-Rahman barostat with a pressure relaxation time of 2ps[8]. Before production runs, all system were subjected to a 5000-step steepest

descent energy minimization to remove bad contacts[9]. After minimization, the systems were heated up to 310K under constant temperature-volume ensemble (NVT). The simulations were saved every 200ps for analysis. The protein, Mg^{2+} ion, and nucleotide were subjected to positional constraints under a harmonic force constant of 1000 kJ/(mol*nm^2) during the NVT step while solvent molecules were free to move. The systems then were further equilibrated using constant pressure ensemble (NPT), in which the force constant applied to the protein, Mg^{2+} ion, and nucleotide were gradually reduced from 5kJ/(mol*nm^2) to zero in six steps of 5ns each. An additional 50ns of unconstraint simulation was performed, making it a total of 80ns NPT equilibration prior to production runs. We performed five production runs of 1000ns for a total of 5000ns.

Root-mean-square-deviation-based (RMSD) conformational clustering

The last 600ns of each production run was concatenated into one trajectory and skipped to keep one frame for every ten frames. Due to the high fluctuation observed in the C terminus (residue 180-216), these residues were excluded from clustering. GROMACS cluster module was used to perform the clustering, with a RMSD cutoff of 0.08nm. C_α atoms of residue 8-179 were used for superposition and RMSD calculation. The top five clusters were selected for further analysis.

FindBindSite

FindBindSite is a method developed to identify druggable small-molecule binding sites in a protein structure[10]. Briefly, we docked a diverse small-molecule library to the entire protein structure and identify the regions that has high docked ligand densities. These high-density regions were then clustered into separate pockets and each pocket was ranked based on the average interaction strength (sum of van der Waals and hydrogen bond energies) of the bound ligands with Ran. The pocket that is formed by the two switches were selected for virtual ligand screening.

Small molecule library preparation and virtual ligand screening (VLS) pipeline

Small molecule libraries were obtained from websites of vendors. For the initial screening, the Core and Express libraries from Chembridge were used (Chembridge, accessed Aug. 2021). Small molecule libraries were prepared using Maestro LigPrep module. The target pH range was set to 7.0±1.0 using Epik. Computation was set to “Generate all combinations” and at most 64 per ligand. Protein grid was generated using Maestro Receptor Grid Generation module. The geometric center of the chosen FBS pocket was supplied as the center of the grid box, and the center of mass box size was reduced to 7Å x 7Å x 7Å. Ligand docking was performed using Maestro Glide module. At most five poses were written to the output, and the rest of the settings were default. After docking, the top 1500 ligands by docking score were selected for further process. Sidechain prediction was applied to residues within 7Å of ligands in the protein-ligand complexes, followed by a global minimization using gradient descent algorithms with a convergence cutoff of 0.1kcal/mol/Å using Maestro Prime module. Protein-ligand contacts were calculated using GetContacts (https://getcontacts.github.io/) between ligands and residues 37-45 in switch I region and residues 65-72 in switch II region. Ligands that did not make contacts with both switch regions were filtered out. MM-GBSA energy between protein and ligands was measured with a nearest neighbor cutoff of 7Å using Prime MM-GBSA module. The energy measured using MM-GBSA was used as the final score for ligands.

Ligand analog search

Search library was obtained from Chembridge (Chembridge, accessed Aug. 2022), MCule(12) PubChem(13), and Enamine (Enamine, accessed Aug. 2022). Ligand analog search was performed in Python environment using RDKit package. The selected candidate was imported and featurized using Morgan Fingerprint. Subsequently, the Tanimoto coefficient of ligands from the search library against the candidate was calculated and ranked. Ligands with Tanimoto coefficient larger or equal to 0.3 was selected for VLS.

Nuclear Magnetic Resonance (NMR)

For saturation transfer difference (STD) NMR study, the molar ratio of compound MAR-3.6.2 vs. RAN is 50 :1 in which the concentration of Ran is 1 μM. The D2O-based 30 mM Sodium phosphate buffer with pH 7.5 was used with 1% DMSO-d6 and 2μM. 20 μM 3-(Trimethylsilyl)-propionic-2,2,3,3,-d4 acid sodium (TSP-d4 from Sigma-Aldrich) was used as internal reference.

NMR STD[11] experiments were carried out at 25°C on 700 MHz Bruker Ascend equipped with 5 mm triple resonance cryogenic probe. The spectral width is 14 ppm with 32k data points. The saturation frequency was set at -0.2 ppm, and the reference experiment frequency was set at -30 ppm. The 50 ms gauss pulse saturation train is 4 second long with field strength of 86 Hz. The T2 filter spin-lock is 70 ms with field strength of 4960 Hz to eliminate residual protein signals. The total number of scan is 4480, and the saturation and reference experiments were acquired in interleaved manner using Bruker stddiffgp19.3 pulse sequence. The data were analyzed using Bruker topspin 3.6. Figure 3A displays the 1D reference spectrum (blue) and the saturated spectrum (red), showing five aromatic protons (top) and three methyl groups (bottom). The green spectrum represents the difference between the reference and saturated spectra. The vertical scale of the bottom figure is set to half of the top figure for ease of visualization.

Saturation Transfer Difference (STD) values (Fig. 3B) were calculated using the formula $(I_{ref} - I_{sat})/I_{ref}$, where the I_{ref} and I_{sat} represent the peak intensities in the reference and saturated spectra, respectively. The STD error was estimated from the noise intensity in the 10–11 ppm spectral range. The STD values and chemical shift values are listed in Fig. 3B. Proton assignments are depicted in the chemical structure of MAR-3.6.2 (Fig. 3A, bottom), where protons 1 and 2, as well as 3 and 4, remain unassigned. Some protons exhibit two distinguishable peaks due to different diastereomers, with the minor and major peaks labeled as (a) and (b), respectively. All aromatic protons and one methyl group attached to the benzyl ring in compound MAR-3.6.2 exhibit significant STD values, indicating direct interaction between MAR-3.6.2 and the RAN protein. The

variation in STD values among protons suggests that the interaction between MAR-3.6.2 and RAN is highly specific[12].

Phalloidin staining

Cells were fixed in 4% paraformaldehyde for 15 minutes at room temperature, followed by permeabilization with 0.05% Triton X-100 for 15 minutes. After washing with PBS three times, cells were stained with Phalloidin-iFluor 488 (Abcam, #ab176753, 1:1000 dilution) in PBS containing 1% BSA for 30 minutes at room temperature in the dark. Stained cells were washed and mounted with ProLong Gold Antifade Mountant (Thermo Fisher). Images were acquired using a Zeiss LSM880 confocal microscope with a 63× oil immersion objective lens, and surface rendering and analysis were performed using Imaris software.

Wound Healing Assay

HUVEC cells were seeded in 6-well plates and grown to ~90% confluency. A uniform scratch was created using a scratcher, and detached cells were removed by washing with PBS. Images were captured at 0, 24, and 48 hours post-scratch using a Zeiss LSM880 confocal microscope. The results were expressed as the levels of wound closure relative to the initial scratch width.

Podosome formation

HUVEC cells were seeded on glass coverslips in a 24-well plate and cultured to ~30% confluency. Cells were fixed with 4% paraformaldehyde for 15 minutes, permeabilized with 0.05% Triton X-100 for 15 minutes, and blocked with 2% BSA in PBS for 30 minutes at room temperature. Cells were then incubated with anti-MT-MMP1 (Santa Cruz, #sc077397, 1:1000) and anti-ACTIN (Santa Cruz, #sc47778, 1:1000) antibodies overnight at 4°C. After washing with PBS, cells were incubated with fluorescent secondary antibodies (1:1000) for 1 hour at room temperature in the dark. Coverslips were mounted using ProLong Gold Antifade Mountant (Thermo Fisher), and

images were acquired using a Zeiss LSM880 confocal microscope. Podosomes were identified as black voids within the ACTIN network.

Gelatin Degradation

HUVEC cells were seeded on fluorescently labeled gelatin-coated coverslips prepared using the Gelatin Degradation Assay Kit (Abcam, #ab234620) according to the manufacturer's protocol. Briefly, coverslips were coated with a gelatin matrix, crosslinked with 0.5% glutaraldehyde, and quenched with 70% ethanol before cell seeding. After treatment, cells were fixed with 4% paraformaldehyde for 15 minutes, permeabilized with 0.05% Triton X-100 for 15 minutes, and blocked with 2% BSA in PBS for 30 minutes. Cells were then stained with anti-VE-Cadherin (Santa Cruz, #sc52751, 1:200) and DAPI for nuclear visualization. Images were acquired using a Zeiss LSM880 confocal microscope; surface rendering and analysis were performed using Imaris software, with gelatin degradation areas identified as black voids within the green gelatin matrix.

3D Sprouting Angiogenic Analysis

To evaluate angiogenic sprouting, a 3D sprouting angiogenesis assay was conducted using AIM Biotech's 3D Cell Culture Chips. Initially, the central gel channel of each chip was filled with a collagen matrix, which was allowed to polymerize to provide a scaffold for cell growth. Subsequently, HUVEC cells were seeded into the adjacent media channels at a density of 1.5 million cells per milliliter. The cells were cultured under standard conditions until a confluent monolayer formed along the gel interface. To induce angiogenic sprouting, a gradient of angiogenic factors, such as Vascular Endothelial Growth Factor (VEGF) and Sphingosine-1-Phosphate (S1P), was established across the gel by adding media with differing concentrations to the media channels. This setup facilitated the formation of new vascular sprouts into the 3D matrix. The dynamic process of sprouting angiogenesis was monitored over 2–3 days using a

Zeiss LSM880 confocal microscope, with detailed images captured for subsequent analysis. Surface rendering and analysis were performed using Imaris software.

PI3K Lipid Assay

1 mg of total protein from treated cells was immunoprecipitated using anti-PI3K p85 antibody (Cell Signaling Technology, #4292) and incubated overnight at 4°C with Protein A/G agarose beads (Santa Cruz, #sc-2003). The immunoprecipitates were washed with lysis buffer and incubated with phosphatidylinositol substrate (Echelon Biosciences, #P-3016) in kinase reaction buffer (50 mM HEPES, 10 mM $MgCl_2$, 1 mM DTT, 0.2 mM ATP, pH 7.4) at 30°C for 30 minutes. The reaction was terminated by the addition of stop buffer (20 mM EDTA). The reaction products were separated by thin-layer chromatography (TLC) on silica plates, and phosphoinositide production was visualized by autoradiography using [γ-^{32}P]ATP (PerkinElmer, #BLU502A250UC). The signal was detected using enhanced chemiluminescence (ECL, Thermo Fisher Scientific).

RNA Sequencing

For comprehensive genomic profiling of mRNA transcripts (mRNA-seq), sequencing was conducted on an Illumina Hiseq 2500 platform. To preprocess the reads, Trimmomatics was employed to trim poly(A) tails and Illumina adapters. Subsequently, alignment to the Human Genome Assembly GRCh38.p14 was performed using Bowtie2 v2.5.1 with default parameters. The expression levels of ensemble genes were quantified using RSEM v1.3.3. Custom R scripts and Bioconductor packages such as "edgeR" were utilized for data normalization and inter-group comparisons. Gene Set Enrichment Analysis (GSEA) v4.3.2 was employed to identify alterations in Gene Ontology (GO) terms and canonical pathways (downloaded from Msigdb v7.0) following treatment with MAR-3.6.2 compared to DMSO control.

Supplementary Reference

1 Jo, S., Kim, T., Iyer, V. G. & Im, W. CHARMM-GUI: a web-based graphical user interface for CHARMM. *J Comput Chem* **29**, 1859-1865, doi:10.1002/jcc.20945 (2008).

2 Hess, B., Kutzner, C., van der Spoel, D. & Lindahl, E. GROMACS 4: Algorithms for Highly Efficient, Load-Balanced, and Scalable Molecular Simulation. *Journal of Chemical Theory and Computation* **4**, 435-447, doi:10.1021/ct700301q (2008).

3 Huang, J., Rauscher, S., Nawrocki, G., Ran, T., Feig, M., de Groot, B. L., Grubmüller, H. & MacKerell, A. D. CHARMM36m: an improved force field for folded and intrinsically disordered proteins. *Nature Methods* **14**, 71-73, doi:10.1038/nmeth.4067 (2017).

4 Berendsen, H. J. C., Postma, J. P. M., van Gunsteren, W. F., DiNola, A. & Haak, J. R. Molecular dynamics with coupling to an external bath. *The Journal of Chemical Physics* **81**, 3684-3690, doi:10.1063/1.448118 (1984).

5 Andersen, H. C. Rattle: A “velocity” version of the shake algorithm for molecular dynamics calculations. *Journal of Computational Physics* **52**, 24-34, doi:https://doi.org/10.1016/0021-9991(83)90014-1 (1983).

6 Darden, T., York, D. & Pedersen, L. Particle mesh Ewald: An N·log(N) method for Ewald sums in large systems. *The Journal of Chemical Physics* **98**, 10089-10092, doi:10.1063/1.464397 (1993).

7 Evans, D. J. & Holian, B. L. The Nose–Hoover thermostat. *The Journal of Chemical Physics* **83**, 4069-4074, doi:10.1063/1.449071 (1985).

8 Parrinello, M. & Rahman, A. Polymorphic transitions in single crystals: A new molecular dynamics method. *Journal of Applied Physics* **52**, 7182-7190, doi:10.1063/1.328693 (1981).

9 Petrova, S. S. & Solov'ev, A. D. The Origin of the Method of Steepest Descent. *Historia Mathematica* **24**, 361-375, doi:https://doi.org/10.1006/hmat.1996.2146 (1997).

10 Li, H., Kasam, V., Tautermann, C. S., Seeliger, D. & Vaidehi, N. Computational Method To Identify Druggable Binding Sites That Target Protein–Protein Interactions. *Journal of Chemical Information and Modeling* **54**, 1391-1400, doi:10.1021/ci400750x (2014).

11 Mayer, M. & Meyer, B. Group Epitope Mapping by Saturation Transfer Difference NMR To Identify Segments of a Ligand in Direct Contact with a Protein Receptor. *Journal of the American Chemical Society* **123**, 6108-6117, doi:10.1021/ja0100120 (2001).

12 Cala, O. & Krimm, I. Ligand-Orientation Based Fragment Selection in STD NMR Screening. *Journal of Medicinal Chemistry* **58**, 8739-8742, doi:10.1021/acs.jmedchem.5b01114 (2015).

Supplementary Table S1. Characteristics of patient samples

Sample ID	Sex	Sample Type	Disease Status	Cytogenetic	Other Mutation	Blasts PB (%)	Blasts BM (%)	Age
AML-1	F	PBMC	AML	47,XX,+21[19];47,sl,del(9)(q?13q22)[3]	CEBPA (c.232del; p.Leu78Trpfs*82); (c.913_921dup; p.Gln305_Asn307dup)	89%	94%	54
AML-2	F	PBMC	AML	Normal	ASXL1 (c.1867C>T; p.Q623*) (50%) BAALC CCND3 CDK6 FLT3 IDH2 (c.419G>A; p.R140Q) (46%) MPL (c.1544G>T; p.W515L) (90%) RUNX1 (c.592G>A; p.D198N) 47% SPARC SRSF2 (c.284C>A; p.P95H) (49%)	80%	96%	74
AML-3	F	PBMC	AML	Normal	DNMT3A (c.2141C>G; p.S714C) (44%) FLT3-ITD IDH2 (c.419G>A; p.R140Q) (45%) NPM1 (c.860_863dupTCTG; p.W288Cfs*12)	90%	>95%	62

AML-4	M	PBMC	AML	46,XY[21]	DNMT3A (c.2645G>A; p.R882H) (44%) FLT3 (c.2503G>T; p.D835Y) (32%) NPM1 (c.863_864insCATG; p.W288Cfs*12) NRAS (c.35G>A; p.G12D) (13%) SMC3 (c.1985G>T; p.G662V) (27%)	62%	45%	46
AML-5	M	PBMC	AML	t(16;16), trisomy 21, trisomy 22	FLT-3 ITD Neg., FLT-3 D835 Pos., NPM1 Neg., C-kit Neg	76%		64

Supplementary Table S2. List of Antibodies used for WB, IP, and flow cytometric analysis

No	Antibody Name	Information
1	APC anti-mouse CD45.2 antibody	1:100 (BioLegend, Cat# 109814)
2	FITC anti-mouse CD45.1 antibody	1:100 (BioLegend, Cat# 110706)
3	APC Annexin V	1:100 (BioLegend, Cat# 640941)
4	Anti-SPRED1 antibody	1:1000 (Santa Cruz Biotechnology, Cat# sc-101392)
5	Anti-RhoA antibody	
6	Anti-PCNA antibody	1:1000 (Santa Cruz Biotechnology, Cat# sc-56)
7	Anti-ACTIN antibody	1:1000 (Santa Cruz Biotechnology, Cat# sc-47778)
8	Anti-p-AKT antibody	1:1000 (Cell Signaling Technology, Cat# 9275S)
9	Anti-PIP2 antibody	
10	Anti-Cortactin antibody	1:1000 (Santa Cruz Biotechnology, Cat# sc-55579)
11	Anti-TSK5 antibody	1:1000 (MilliporeSigma, Cat# 09-403)
12	Anti-RAN antibody	1:1000 (Santa Cruz Biotechnology, Cat# sc-271376)
13	Anti-PARP antibody	1:1000 (Cell Signaling Technology, Cat# 9452S)
14	Anti-XPO5 antibody	1:1000 (Cell Signaling Technology, Cat# 12565S)
15	Anti-RCC1 antibody	1:1000 (Santa Cruz Biotechnology, Cat# sc-1161)
16	Anti-MT-MMP1	1:1000 (Santa Cruz Biotechnology, Cat# sc077397)
17	Anti-VE-Cadherin	1:200 (Santa Cruz Biotechnology, Cat# sc52751)
18	Anti-PI3K p85 antibody	1:1000 (Cell Signaling Technology, Cat#4292)
19	Protein A/G agarose beads	Santa Cruz Technology, Cat# sc2003
20	Anti-Rab5 (C8B1) antibody	1:1000 (Cell Signaling Technology, Cat# 3547T)

21	Anti-RAS antibody	1:1000 (Cell Signaling Technology, Cat# 3965S)
22	Anti-NUP358 antibody	1:1000 (ThermoFisher, Cat# MA1-847)

Table S3. Molecular dynamics simulation setup detail

Total number of atoms	49714
Total protein atoms	3449
Total ligand atoms	41
Total number of water molecules	15379
Salt concentration	0.15M
Box dimension	7.5nmx7.5nmx7.5nm

Supplementary Figure and Legends

A

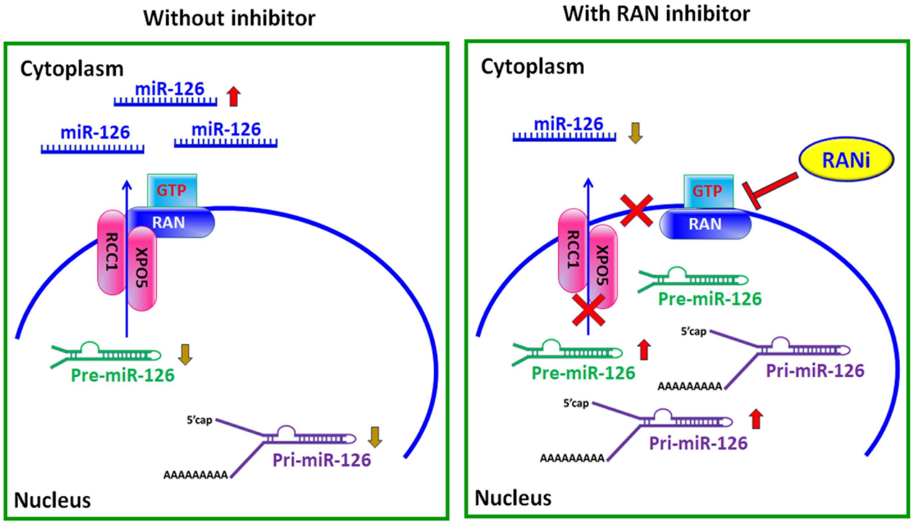

Without inhibitor
With RAN inhibitor
Cytoplasm
miR-126
GTP
RAN
RCC1
XPO5
Pre-miR-126
5'cap
Pri-miR-126
AAAAAAAAA
Nucleus
RANi

B

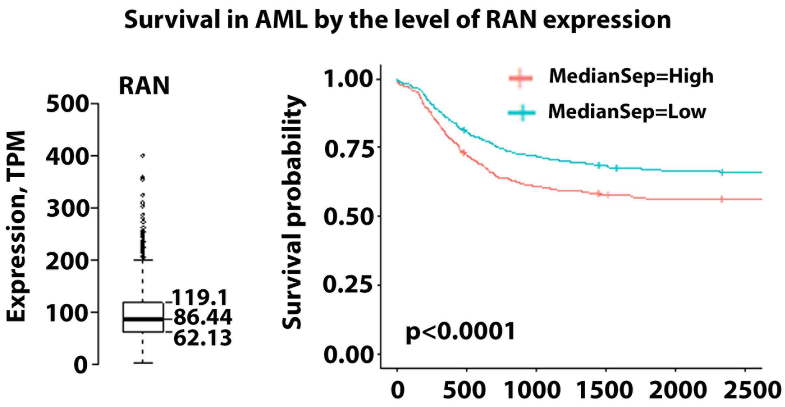

Survival in AML by the level of RAN expression
RAN
Expression, TPM
500
400
300
200
100
0
119.1
86.44
62.13
Survival probability
1.00
0.75
0.50
0.25
0.00
MedianSep=High
MedianSep=Low
p<0.0001
0
500
1000
1500
2000
2500

Figure S1

Figure S1. Effects of RAN on regulation of miR-126 biogenesis. **A** Schematic model of RAN-regulated miR-126 biogenesis. Left, in the absence of a RAN inhibitor, RAN-GTP binds to RCC1 and XPO5, facilitating the export of pre-miR-126 from the nucleus to the cytoplasm. This results in decreased levels of pre- and pri-miR-126 and an increase in mature miR-126. Right, in the presence of a RAN inhibitor, the interaction between RAN, RCC1, and XPO5 is disrupted, impairing the transport of pre-miR-126 to the cytoplasm. This leads to an increase in pre- and pri-miR-126 and a decrease in mature miR-126. **B** Kaplan-Meier survival analysis of AML patients based on RAN expression levels. Left, RAN expression levels from the TARGET AML dataset. Right, Kaplan-Meier survival analysis of the TARGET dataset, showing the correlation between RAN expression levels and overall survival in AML patients.

A

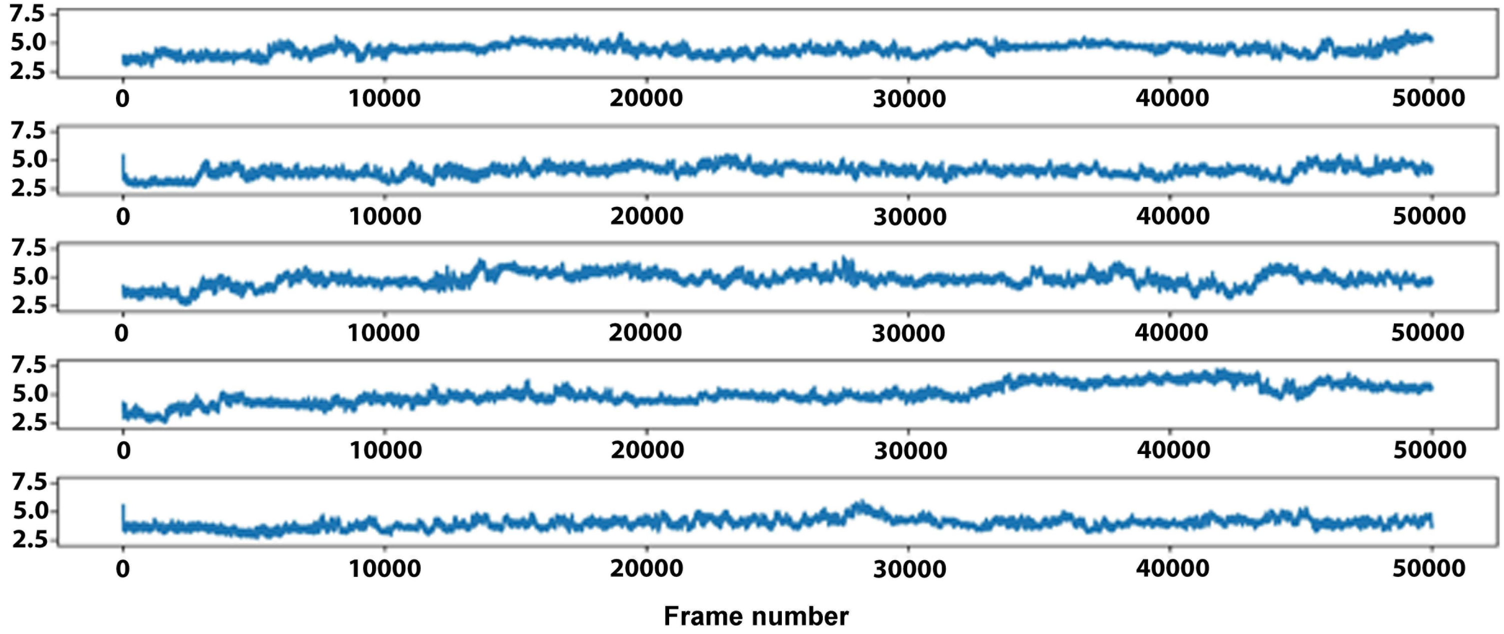
7.5
5.0
2.5
0
10000
20000
30000
40000
50000
Frame number

B

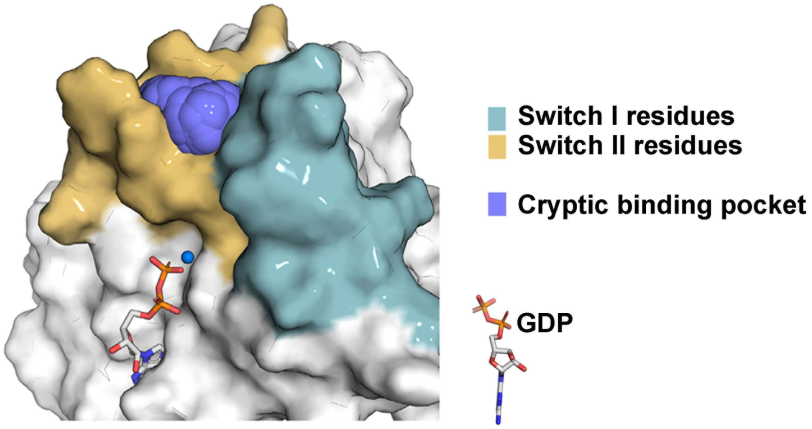
Switch I residues
Switch II residues
Cryptic binding pocket
GDP

Figure S2

Figure S2. Cryptic binding pocket of RAN protein. A RMSD of the protein Cα atoms during the MD simulation. RMSD of backbone atoms relative to the initial minimized structure is shown as a function of simulation frames. **B** Location of the cryptic binding pocket identified between switch I and II regions.

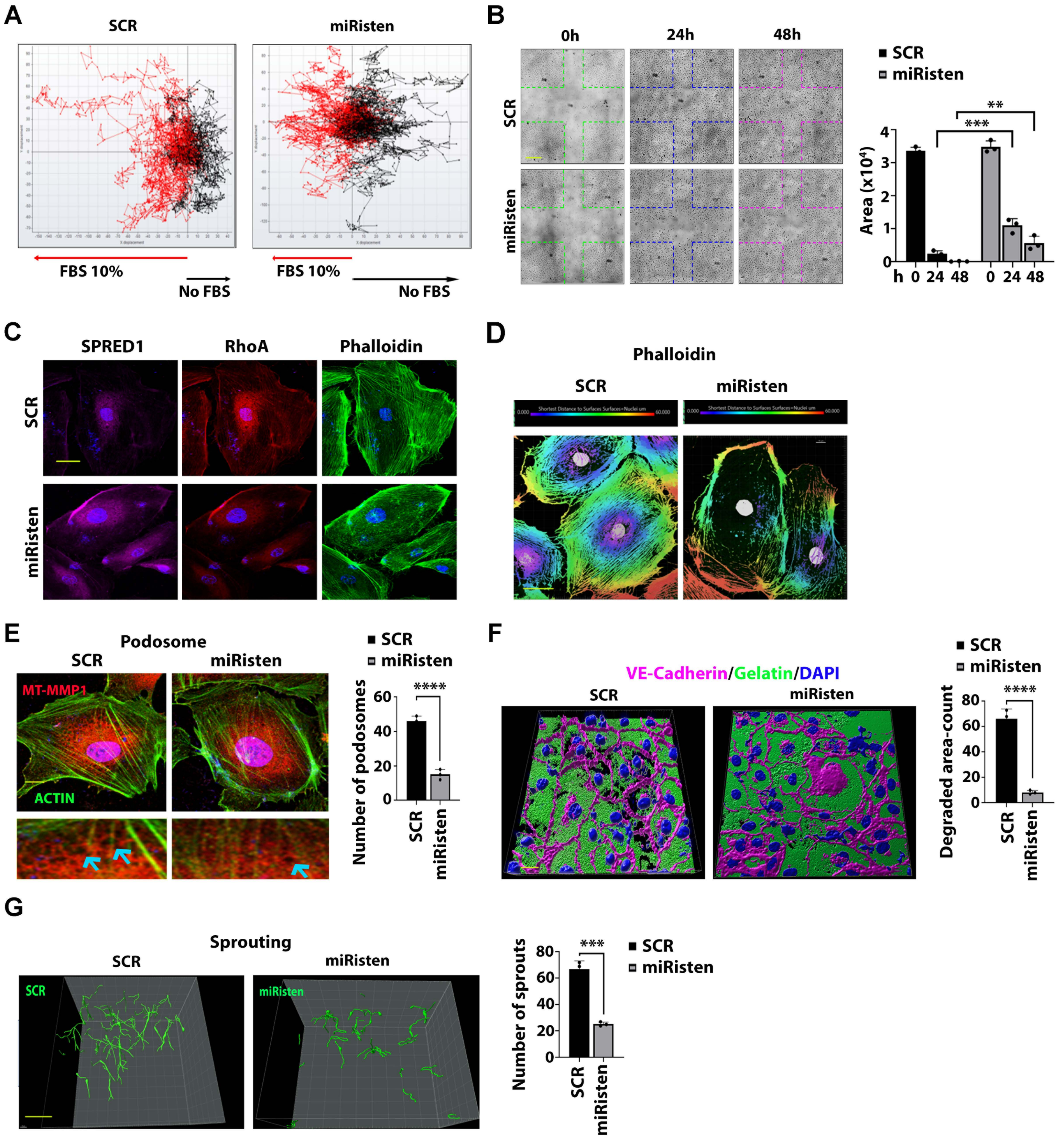
A
SCR
miRisten
FBS 10%
No FBS
FBS 10%
No FBS
B
0h
24h
48h
SCR
miRisten
SCR
miRisten
Area (x10⁴)
***
**
h 0 24 48 0 24 48
C
SPRED1
RhoA
Phalloidin
SCR
miRisten
D
Phalloidin
SCR
miRisten
E
Podosome
SCR
miRisten
MT-MMP1
ACTIN
SCR
miRisten
Number of podosomes
****
F
VE-Cadherin/Gelatin/DAPI
SCR
miRisten
SCR
miRisten
Degraded area-count
****
G
Sprouting
SCR
miRisten
SCR
miRisten
Number of sprouts
***
SCR
miRisten

Figure S3

Figure S3. Effects of miRisten on endothelial cell activities. A-G HUVEC cells were treated with SCR control or miRisten (2 μM) for 24 hours. **A** Effects of miRisten on chemoattractant cues in HUVEC cells. HUVEC cells treated with SCR control or miRisten (2 μM) were cultured in the central gel channel of AIM Biotech's 3D Cell Culture Chips. The left media channel contained 10% FBS as a chemoattractant cue, while the right media channel contained FBS-free culture medium. Cell migration was recorded using a Zeiss LSM880 confocal microscope, with directional movement represented as red for cells migrating toward the FBS channel and black for cells moving toward the FBS-free side. **B** Effects of miRisten on wound healing activities. A scratch assay was conducted to assess the effects of miRisten on wound healing activity in HUVEC cells. Cells were treated with SCR control or miRisten (2 μM) for 24 hours prior to scratch induction. Images were captured at the indicated time points post-scratch using a confocal microscope. Scale bar, 200 μM. Quantification shown on the right. **C** Treated cells were stained with anti-SPRED1 (pink), anti-RhoA (red) antibodies, and Phalloidin (green). Scale bar, 10 μM. **D** Representative 3D confocal microscopy images of treated cells stained with Phalloidin. Surface analysis of Phalloidin signal was performed using Imaris software. Scale bar, 20 μM. **E** Representative 3D images of treated cells stained with anti-MT-MMP1 and anti-ACTIN antibodies. Scale bar, 10 μM. Magnified images are shown at the bottom. Podosomes (black holes) are indicated by arrowheads. Quantification shown on the right. **F** Treated cells were cultured on a gelatin-coated layer (green) and stained with anti-VE-Cadherin (pink) and DAPI (blue). Scale bar, 50 μM. Gelatin degradation areas (black voids) are indicated by arrowheads. Surface analysis of fluorescence signals was performed using Imaris software. Quantification shown on the right. **G** Treated cells were assessed for new vascular sprout growth in a 3D matrix from a pre-existing endothelial monolayer (see methods). Images were acquired using confocal microscopy and surface analysis was conducted with Imaris software. Scale bar, 200 μM. Quantification shown

on the right. Data are mean ± SE, based on triplicate determinations, and presented in a bar graph.

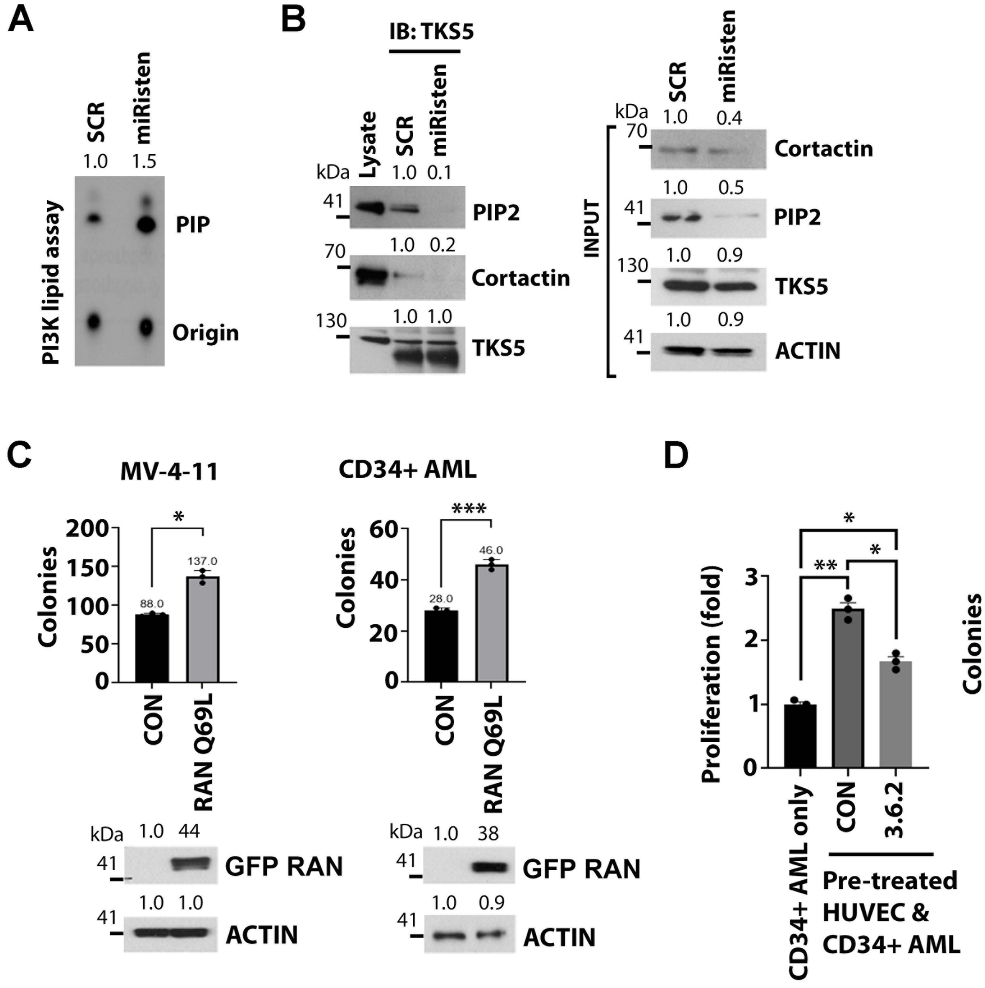
A
SCR
miRisten
1.0
1.5
PI3K lipid assay
PIP
Origin
B
IB: TKS5
Lysate
SCR
miRisten
kDa
1.0
0.1
41
PIP2
70
1.0
0.2
Cortactin
130
1.0
1.0
TKS5
INPUT
SCR
miRisten
kDa
1.0
0.4
70
Cortactin
1.0
0.5
41
PIP2
1.0
0.9
130
TKS5
1.0
0.9
41
ACTIN
C
MV-4-11
CD34+ AML
Colonies
CON
RAN Q69L
GFP RAN
ACTIN
D
Proliferation (fold)
Colonies
miR-126/RNU44 (fold)
CD34+ AML only
CON
3.6.2
Pre-treated HUVEC & CD34+ AML
ns

Figure S4

Figure S4. miRisten effect on endothelial cell activity and anti-leukemic effect of RAN inhibitor MAR-3.6.2 in vitro. A-B Effects of miRisten on PIP signaling. **A** Phosphoinositide (PIP) production was analyzed using a PI3K lipid assay. **B** Interaction of PIP2 and Cortactin with TSK5, with input loading control. **C-D** Anti-leukemic effect of RAN inhibitor MAR-3.6.2. **C** Effect of MAR-3.6.2 on colony formation of active RAN mutant AML cells. MV4-11 and CD34+ AML cells were transfected with SCR control or RAN-Q69L overexpression plasmid. 2×10^5 cells/mL (n = 3) were treated with MAR-3.6.2 (25 μM) for 24 hours, then plated in methylcellulose. After 14 days, colonies were counted (top). Data are mean ± SE, based on triplicate determinations, and presented in a bar graph. Immunoblotting of RAN Q69L overexpression are shown. **D** Effect of MAR-3.6.2 on LSC and HUVEC cell co-culture. 3×10^4 HUVEC cells were seeded into wells of a 24-well plate overnight and treated with DMSO control or MAR-3.6.2 for 24 hours. 3×10^5 CD34+ AML cells were added to treated HUVEC cells. After 24 hours, CD34+ AML cells were collected for proliferation analysis measured by WST-1 (left) and mature miR-126 analysis measured by qPCR (right). 1×10^4 CD34+ AML cells were collected from co-culture and plated in methylcellulose, after 14 days, colonies were counted (middle). Data are mean ± SE, based on triplicate determinations, and presented in a bar graph.
