# Supplementary material for: Allosteric inhibition of RAN decreases miR-126 biogenesis in endothelial cells and controls acute myeloid leukemia growth
Source: Commun Biol. 2026 Apr 14;9:791. doi: 10.1038/s42003-026-10026-0 (PMC13254346; doi:10.1038/s42003-026-10026-0)
Supplement: Supplementary file 2 — Description of Additional Supplementary Files [file 42003_2026_10026_MOESM2_ESM.pdf]

Description of Additional Supplementary Files

File name: Supplementary Data 1

Description: Source data, including uncropped western blots and numerical values, are provided in Supplementary Data 1.
