# Supplementary material for: Allosteric inhibition of RAN decreases miR-126 biogenesis in endothelial cells and controls acute myeloid leukemia growth
Source: Commun Biol. 2026 Apr 14;9:791. doi: 10.1038/s42003-026-10026-0 (PMC13254346; doi:10.1038/s42003-026-10026-0)
Supplement: Supplementary file 4 — Reporting Summary [file 42003_2026_10026_MOESM4_ESM.pdf]

nature portfolio

Corresponding author(s): Nagarajan Vaidehi, Le Xuan Truong Nguyen, Guido Marcucci

Last updated by author(s): Mar 27, 2026

Reporting Summary

Nature Portfolio wishes to improve the reproducibility of the work that we publish. This form provides structure for consistency and transparency in reporting. For further information on Nature Portfolio policies, see our Editorial Policies and the Editorial Policy Checklist.

Statistics

For all statistical analyses, confirm that the following items are present in the figure legend, table legend, main text, or Methods section.

n/a	Confirmed	
☐	☒	The exact sample size (*n*) for each experimental group/condition, given as a discrete number and unit of measurement
☐	☒	A statement on whether measurements were taken from distinct samples or whether the same sample was measured repeatedly
☐	☒	The statistical test(s) used AND whether they are one- or two-sided<br>*Only common tests should be described solely by name; describe more complex techniques in the Methods section.*
☒	☐	A description of all covariates tested
☒	☐	A description of any assumptions or corrections, such as tests of normality and adjustment for multiple comparisons
☐	☒	A full description of the statistical parameters including central tendency (e.g. means) or other basic estimates (e.g. regression coefficient) AND variation (e.g. standard deviation) or associated estimates of uncertainty (e.g. confidence intervals)
☐	☒	For null hypothesis testing, the test statistic (e.g. *F*, *t*, *r*) with confidence intervals, effect sizes, degrees of freedom and *P* value noted<br>*Give P values as exact values whenever suitable.*
☒	☐	For Bayesian analysis, information on the choice of priors and Markov chain Monte Carlo settings
☒	☐	For hierarchical and complex designs, identification of the appropriate level for tests and full reporting of outcomes
☒	☐	Estimates of effect sizes (e.g. Cohen's *d*, Pearson's *r*), indicating how they were calculated

Our web collection on statistics for biologists contains articles on many of the points above.

Software and code

Policy information about availability of computer code

Data collection	n/a
Data analysis	n/a

For manuscripts utilizing custom algorithms or software that are central to the research but not yet described in published literature, software must be made available to editors and reviewers. We strongly encourage code deposition in a community repository (e.g. GitHub). See the Nature Portfolio guidelines for submitting code & software for further information.

Data

Policy information about availability of data

All manuscripts must include a data availability statement. This statement should provide the following information, where applicable:

- Accession codes, unique identifiers, or web links for publicly available datasets
- A description of any restrictions on data availability
- For clinical datasets or third party data, please ensure that the statement adheres to our policy

The RNA-seq data reported in this article has been deposited in NCBI's Gene Expression Omnibus (GEO) and is accessible through GEO Series accession number GSE324546. All molecular dynamics simulation trajectories, topology files, and analysis scripts generated in this study are available at the MDRepo website, https://mdrepo.org, through the ID number MDR00021342. Source data, including uncropped western blots and numerical values, are provided in Supplementary Data 1. All other datasets generated during this study are available from the corresponding author on reasonable request.

Research involving human participants, their data, or biological material

Policy information about studies with human participants or human data. See also policy information about sex, gender (identity/presentation), and sexual orientation and race, ethnicity and racism.

Reporting on sex and gender	Both male and female were consdered in our study, and detailed information is provided in Table S1
Reporting on race, ethnicity, or other socially relevant groupings	N/A
Population characteristics	All age and disease type information is provided in Table S1
Recruitment	Patient samples were recruited based on disease type, with details provided in the Methods section.
Ethics oversight	The procedures were reviewed and approved by the City of Hope Institutional Review Board, following an assurance filed with and approved by the Department of Health and Human Services.

Note that full information on the approval of the study protocol must also be provided in the manuscript.

Field-specific reporting

Please select the one below that is the best fit for your research. If you are not sure, read the appropriate sections before making your selection.

☒ Life sciences ☐ Behavioural & social sciences ☐ Ecological, evolutionary & environmental sciences

For a reference copy of the document with all sections, see nature.com/documents/nr-reporting-summary-flat.pdf

Life sciences study design

All studies must disclose on these points even when the disclosure is negative.

Sample size	Sample sizes chosen are indicated in the individual figure legends and were not based on formal power calculations to detect prespecified effect sizes.
Data exclusions	no data was exclude in the study
Replication	Results with triplicate measurements were included, unless specified otherwise.
Randomization	Mice were randomly assigned to treatment groups.
Blinding	The investigators were blinded to group allocation during data collection and analysis.

Reporting for specific materials, systems and methods

We require information from authors about some types of materials, experimental systems and methods used in many studies. Here, indicate whether each material, system or method listed is relevant to your study. If you are not sure if a list item applies to your research, read the appropriate section before selecting a response.

Materials & experimental systems

n/a	Involved in the study	
☐	☒	Antibodies
☒	☐	Eukaryotic cell lines
☒	☐	Palaeontology and archaeology
☐	☒	Animals and other organisms
☒	☐	Clinical data
☒	☐	Dual use research of concern
☒	☐	Plants

Methods

n/a	Involved in the study	
☒	☐	ChIP-seq
☒	☐	Flow cytometry
☒	☐	MRI-based neuroimaging

Antibodies

Antibodies used	1 APC anti-mouse CD45.2 antibody 1:100 (BioLegend, Cat# 109814)<br>2 FITC anti-mouse CD45.1 antibody 1:100 (BioLegend, Cat# 110706)<br>3 APC Annexin V 1:100 (BioLegend, Cat# 640941)

	4 Anti-SPRED1 antibody 1:1000 (Santa Cruz Biotechnology, Cat# sc-101392)<br>5 Anti-RhoA antibody<br>6 Anti-PCNA antibody 1:1000 (Santa Cruz Biotechnology, Cat# sc-56)<br>7 Anti-ACTIN antibody 1:1000 (Santa Cruz Biotechnology, Cat# sc-47778)<br>8 Anti-p-AKT antibody 1:1000 (Cell Signaling Technology, Cat# 9275S)<br>9 Anti-PIP2 antibody<br>10 Anti-Cortactin antibody 1:1000 (Santa Cruz Biotechnology, Cat# sc-55579)<br>11 Anti-TSK5 antibody 1:1000 (MilliporeSigma, Cat# 09-403)<br>12 Anti-RAN antibody 1:1000 (Santa Cruz Biotechnology, Cat# sc-271376)<br>13 Anti-PARP antibody 1:1000 (Cell Signaling Technology, Cat# 9452S)<br>14 Anti-XPO5 antibody 1:1000 (Cell Signaling Technology, Cat# 12565S)<br>15 Anti-RCC1 antibody 1:1000 (Santa Cruz Biotechnology, Cat# sc-1161)<br>16 Anti-MT-MMP1 1:1000 (Santa Cruz Biotechnology, Cat# sc077397)<br>17 Anti-VE-Cadherin 1:200 (Santa Cruz Biotechnology, Cat# sc52751)<br>18 Anti-PI3K p85 antibody 1:1000 (Cell Signaling Technology, Cat#4292)<br>19 Protein A/G agarose beads Santa Cruz Technology, Cat# sc2003
Validation	The species and application details are available on the manufacturer's website.

Animals and other research organisms

Policy information about studies involving animals; ARRIVE guidelines recommended for reporting animal research, and Sex and Gender in Research

Laboratory animals	Cohorts of AML mice were generated via tail vein i.v. injection into sublethally irradiated (X-RAD 320-Precision X-Ray; 4.5 Gy) 6–8 week-old syngenic C57BL/6 wild-type mice (wt, both female and male).
Wild animals	n/a
Reporting on sex	Female mice were considered in our study
Field-collected samples	Mice were maintained on 12-hr light/12-hr dark cycles under 18-23°C ambient temperature with 40-60% humidity in an Association for Assessment and Accreditation of Laboratory Animal Care–accredited animal facility.
Ethics oversight	All experimental procedures were performed following federal and state government guidelines and established institutional guidelines and protocols approved by the Institutional Animal Care and Use Committee at the City of Hope.

Note that full information on the approval of the study protocol must also be provided in the manuscript.

Plants

Seed stocks	N/A
Novel plant genotypes	N/A
Authentication	N/A
